# Jasmonate promotes artemisinin biosynthesis by activating the TCP14-ORA complex in *Artemisia annua*

**DOI:** 10.1126/sciadv.aas9357

**Published:** 2018-11-14

**Authors:** Ya-Nan Ma, Dong-Bei Xu, Ling Li, Fei Zhang, Xue-Qing Fu, Qian Shen, Xue-Ying Lyu, Zhang-Kuanyu Wu, Qi-Fang Pan, Pu Shi, Xiao-Long Hao, Ting-Xiang Yan, Ming-Hui Chen, Pin Liu, Qian He, Li-Hui Xie, Yi-Jun Zhong, Yue-Li Tang, Jing-Ya Zhao, Li-Da Zhang, Xiao-Fen Sun, Ke-Xuan Tang

**Affiliations:** Joint International Research Laboratory of Metabolic and Developmental Sciences, Key Laboratory of Urban Agriculture (South) Ministry of Agriculture, Plant Biotechnology Research Center, Fudan-SJTU-Nottingham Plant Biotechnology R&D Center, School of Agriculture and Biology, Shanghai Jiao Tong University, Shanghai 200240, China.

## Abstract

*Artemisia annua* produces the valuable medicinal component, artemisinin, which is a sesquiterpene lactone widely used in malaria treatment. AaORA, a homolog of CrORCA3, which is involved in activating terpenoid indole alkaloid biosynthesis in *Catharanthus roseus*, is a jasmonate (JA)–responsive and trichome-specific APETALA2/ETHYLENE-RESPONSE FACTOR that plays a pivotal role in artemisinin biosynthesis. However, the JA signaling mechanism underlying AaORA-mediated artemisinin biosynthesis remains enigmatic. Here, we report that AaORA forms a transcriptional activator complex with AaTCP14 (TEOSINTE BRANCHED 1/CYCLOIDEA/PROLIFERATING CELL FACTOR 14), which is also predominantly expressed in trichomes. AaORA and AaTCP14 synergistically bind to and activate the promoters of two genes, *double bond reductase 2* (*DBR2*) and *aldehyde dehydrogenase 1* (*ALDH1*), both of which encode enzymes vital for artemisinin biosynthesis. AaJAZ8, a repressor of the JA signaling pathway, interacts with both AaTCP14 and AaORA and represses the ability of the AaTCP14-AaORA complex to activate the *DBR2* promoter. JA treatment induces AaJAZ8 degradation, allowing the AaTCP14-AaORA complex to subsequently activate the expression of *DBR2*, which is essential for artemisinin biosynthesis. These data suggest that JA activation of the AaTCP14-AaORA complex regulates artemisinin biosynthesis. Together, our findings reveal a novel artemisinin biosynthetic pathway regulatory network and provide new insight into how specialized metabolism is modulated by the JA signaling pathway in plants.

## Teaser

JA-induced JAZ8 degradation releases and activates the TCP14-ORA complex in enhancing artemisinin biosynthesis in *Artemisia annua*.

## INTRODUCTION

Artemisinin, a sesquiterpene lactone, and its semisynthetic derivatives (artemether and artesunate) are famous for their use in the treatment of malaria. To decrease the high risk of drug resistance when using a single drug, such as chloroquine, mefloquine, and sulfadoxine-pyrimethamine, to treat malaria, the use of artemisinin-based combination therapies has been proposed by the World Health Organization (*1*, *2*). In plants, *Artemisia annua* is the only natural source of artemisinin, and the artemisinin content in this plant is very low (0.01 to 1.0% by dry weight) (*3*). The low artemisinin content and unstable supply of plant-derived artemisinin have motivated the semisynthetic production of artemisinin in yeast (*4*, *5*). However, the limited production in engineered yeast cannot meet the high demand for artemisinin production, and *A. annua* plants are still the major suppliers of this compound.

So far, available evidence indicates that the glandular secretory trichomes (GSTs) of *A. annua* are biofactories for artemisinin biosynthesis and accumulation (*6*), and the artemisinin biosynthetic pathway has been well elucidated (fig. S1A) (*4*, *5*, *7*–*13*). Briefly, farnesyl diphosphate synthase (FPS) catalyzes the production of farnesyl diphosphate (FPP) from isopentenyl diphosphate and isomer dimethylallyl diphosphate, which are produced from both the cytosolic mevalonic acid pathway and the plastidial methylerythritol diphosphate pathway (*7*). The first committed step of artemisinin biosynthesis is the cyclization of FPP to amorpha-4,11-diene by amorpha-4,11-diene synthase (ADS) (*8*). Amorpha-4,11-diene is then successively oxidized to yield artemisinic alcohol, artemisinic aldehyde, and artemisinic acid (AA) by cytochrome P450–dependent hydroxylase (CYP71AV1) along with the reduced form of nicotinamide adenine dinucleotide phosphate: cytochrome P450 oxidoreductase or alcohol dehydrogenase 1 (*4*, *5*, *9*). In addition, artemisinic aldehyde is converted to dihydroartemisinic aldehyde by double bond reductase 2 (DBR2) (*10*). Then, aldehyde dehydrogenase 1 (ALDH1) catalyzes the formation of dihydroartemisinic acid (DHAA) from dihydroartemisinic aldehyde (*11*). Last, AA and DHAA may be transported to the trichome subcuticular space and then converted to arteannuin B (AB) and artemisinin by an enzyme-independent reaction (*12*, *13*). It is noteworthy that artemisinic aldehyde is the last common intermediate of the two pathways leading to AB and artemisinin. Consequently, to produce high levels of artemisinin, efficient reduction of artemisinic aldehyde to dihydroartemisinic aldehyde by DBR2 is necessary (*14*).

Although the artemisinin biosynthetic pathway has been elucidated, the transcriptional regulation of this pathway remains largely unknown. Several lines of evidence suggest that transcription factors play pivotal roles in plant specialized metabolism, including in artemisinin biosynthesis (*15*). An *A. annua* WRKY transcription factor, AaWRKY1, was reported to be a positive regulator of *ADS* and *CYP71AV1* expression (*16*). The jasmonate (JA)–responsive *A. annua* GST-specific WRKY1 (AaGSW1) was found to promote artemisinin biosynthesis by directly binding to and activating the *CYP71AV1* promoter (*17*). Artemisinin biosynthesis is positively regulated by the basic helix-loop-helix (bHLH) transcription factor AaMYC2, which promotes the expression of *CYP71AV1* and *DBR2*, and the basic leucine zipper transcription factor AabZIP1 (basic leucine-zipper 1), which promotes the expression of *ADS* and *CYP71AV1* (*18*, *19*). In addition, several APETALA2/ETHYLENE-RESPONSIVE FACTOR (AP2/ERF) proteins, including AaERF1, AaERF2, and AaTAR1, have also been shown to positively regulate artemisinin biosynthesis by up-regulating *ADS* and *CYP71AV1* expression (*20*, *21*). Moreover, *A. annua* octadecanoid–responsive AP2/ERF (*AaORA*) has been shown to be a trichome-specific AP2/ERF transcription factor that activates artemisinin biosynthesis by up-regulating *ADS*, *CYP71AV1*, and *DBR2* expression (*22*). Furthermore, it was recently reported that *AaORA* is a direct target of AaGSW1 and that *AaORA* expression is also up-regulated after JA treatment (*17*), suggesting that an AaORA-related regulatory module is important for JA responses. It is worth mentioning that AaORA is a close homolog of CrORCAs in *C. roseus* and *NIC2*-locus ERFs in *Nicotiana tabacum*, and members of these families play vital roles in the synthesis of terpenoid indole alkaloids (TIAs) and nicotine (*23*, *24*). Likewise, both CrORCA2 and CrORCA3 are downstream components of JA signaling pathways and are regulated by CrMYC2 (*25*). Thus, the results from these previous studies indicate that AP2/ERF proteins are crucial regulators of plant specialized metabolism and hormone signaling. However, except for these few examples, very little is known about AP2/ERF regulatory networks, especially the candidate partners, and the mechanism by which AaORA regulates artemisinin biosynthesis in *A. annua* is completely unknown.

Other transcription factors in addition to those mentioned above might also play important roles in specialized metabolism, such as the members of the TEOSINTE BRANCHED 1/CYCLOIDEA/PROLIFERATING CELL FACTOR (TCP) family. Members of this family, which was discovered in 1999, contain a noncanonical bHLH and are divided into two groups on the basis of the bHLH motif: class I [PCF (proliferating cell nuclear antigen factor) or TCP-P] and class II (TCP-C) (*26*). In *Arabidopsis thaliana*, class I TCP proteins specifically recognize the sequences GGNCCCAC, GGNCC, or GCCCR (R = A or G), and class II TCP proteins specifically recognize the sequence G(T/C)GGNCCC (*27*, *28*). In plants, TCP proteins function as key regulators of plant developmental processes (*29*), hormonal pathways (*30*–*32*), the circadian clock (*33*), and immunity (*34*), and several members of this family play key roles in plant metabolism (*35*, *36*). However, the role of TCP proteins in artemisinin biosynthesis is unknown and needs to be further investigated.

Apart from transcription factors, the plant hormone JA plays a key role in artemisinin biosynthesis. Spraying *A. annua* with methyl JAs (MeJAs) increased artemisinin content by up-regulating the transcription of artemisinin biosynthetic genes and promoting GST formation (*37*). Overexpression of allene oxide cyclase, a key enzyme in JA biosynthesis, increased JA content and promoted artemisinin biosynthesis by up-regulating the expression of *FPS*, *CYP71AV1*, and *DBR2* (*38*). It is worth mentioning that these genes are also up-regulated by overexpression of the JA-responsive transcription factor, *AaORA* (*17*, *22*), suggesting that JA signaling may activate artemisinin biosynthesis by modulating AaORA activity.

JA signaling has been well studied in the model plant *A. thaliana*. Several JASMONATE ZIM-DOMAIN (JAZ) proteins, which are key regulators of JA signaling, act as repressors by recruiting members of the TOPLESS family of transcriptional corepressors through the adaptor protein, Novel Interactor of JAZ (*39*). The JAZ repressors interact with transcriptional activators, such as MYC2, to inhibit their transcriptional activation activity. The activity of the JAZ repressors is regulated, in turn, by the JA receptor, CORONATINE INSENSITIVE 1 (COI1), which is an F-box protein that plays a role in E3 ubiquitin ligase Skp/Cullin/F-box complex (SCF^COI1^)–mediated proteasomal degradation (*40*, *41*). Following the conjugation of JA to isoleucine to form the active hormone JA-isoleucine (JA-Ile), JA-Ile binds to COI1 to facilitate the formation of COI1-JAZ complexes, leading to the ubiquitination and subsequent degradation of the JAZ proteins, which liberates transcriptional activators involved in diverse JA-mediated responses (*41*). In *A. annua*, it was reported that AaJAZ8 repressed the transcriptional activation activity of HOMEODOMAIN PROTEIN 1 (AaHD1), which regulates JA-mediated trichome development and artemisinin content (*42*). Nevertheless, further study is needed to determine whether AaJAZ8 affects the activation of other transcriptional regulators of artemisinin biosynthesis.

In this study, we identified AaTCP14 as a novel interactor of AaORA and demonstrated that AaORA and AaTCP14 form a complex that modulates artemisinin biosynthesis by directly activating the expression of *DBR2* and *ALDH1*. The repressor AaJAZ8 interacts with both AaORA and AaTCP14, which reduces the interaction between AaORA and AaTCP14. Moreover, AaJAZ8 attenuates the activation of *DBR2* by repressing the AaTCP14-AaORA complex. MeJA treatment induces the degradation of AaJAZ8 and liberates the AaTCP14-AaORA complex, leading to activation of the *DBR2* promoter, which subsequently promotes JA-induced artemisinin accumulation. Thus, our research reveals a novel mechanism underlying JA-mediated control of artemisinin biosynthesis.

## RESULTS

### Identification of AaTCP14 as an AaORA-interacting protein

AaORA significantly activated the promoters of four key genes involved in artemisinin biosynthesis, *ADS*, *CYP71AV1*, *DBR2*, and *ALDH1*, in dual-luciferase (LUC) assays (fig. S1B), which is consistent with the positive role of AaORA in artemisinin biosynthesis (*22*). However, interactions between AaORA and the promoters of these genes were not detected in yeast one-hybrid (Y1H) assays (fig. S1C), implying that AaORA might fulfill its positive regulatory function, in part, by interacting with other DNA-binding transcription factors.

**Fig. 1:**
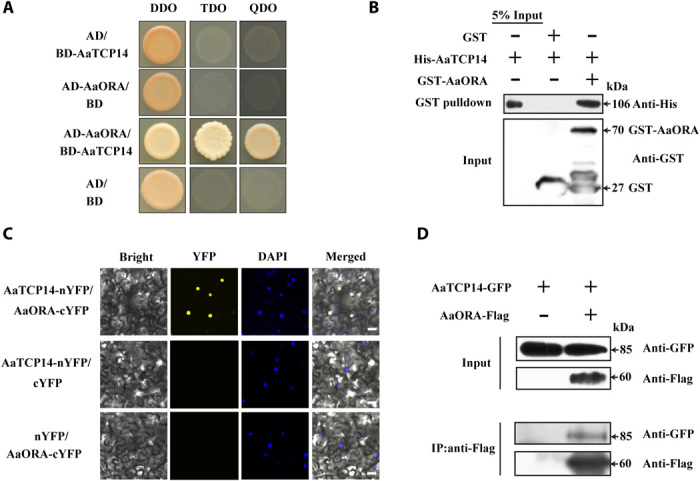
AaTCP14 protein interacts with AaORA. (**A**) Y2H analysis of AaTCP14 interaction with AaORA. Yeast cells transformed with different combinations of constructs containing AaTCP14 fused with the DNA binding domain (BD-AaTCP14), AaORA fused with the activation domain (AD-AaORA), the BD alone, and the AD alone were grown on two different selective media, SD/-Trp/-Leu/-His (TDO) and SD/-Trp/-Leu/-His/-Ade (QDO), and the control medium SD/-Trp/-Leu (DDO). Pictures were taken after 4 days of incubation at 30°C. Y2H assays were repeated three times, and representative results are shown. (**B**) In vitro pulldown assays of AaTCP14 and AaORA recombinant proteins. His-AaTCP14 proteins were pulled down with GST-AaORA and further detected on Western blots probed with anti-His antibody. Experiments were carried out three times, and representative results are shown. (**C**) Bimolecular fluorescence complementation (BiFC) analysis of the interaction between AaTCP14 and AaORA in *N*. *benthamiana* cells. AaTCP14 was fused to the N-terminal fragment of yellow fluorescent protein (AaTCP14-nYFP), and AaORA was fused to the C-terminal fragment of YFP (AaORA-cYFP). Colocalization of reconstituted YFP and nuclei was determined by 4′,6-diamidino-2-phenylindole (DAPI) staining. Three independent transfection experiments were performed. Scale bars, 20 μm. (**D**) Co-IP studies of AaTCP14 and AaORA complex formation in *N. benthamiana* leaves. Total protein extracts from *N. benthamiana* leaves infiltrated with constructs harboring AaTCP14-GFP and AaORA-Flag were immunoprecipitated with anti-Flag antibody. The coimmunoprecipitated proteins were detected by anti-GFP antibody. Experiments were repeated three times and similar results were obtained.

To identify putative AaORA-interacting transcription factors and to elucidate the molecular basis for AaORA control of artemisinin biosynthesis, a Y2H screen for AaORA-interacting proteins was performed using an *A. annua* complementary DNA (cDNA) library constructed from the youngest leaves and meristem. Deletion analysis revealed that full-length AaORA had autonomous transcriptional activation activity (fig. S2); therefore, a truncated version of the AaORA protein, AaORAΔN1 (C terminus of AaORA), lacking autonomous transcriptional activation activity was used as the bait. An initial screen identified AaTCP14, a TCP transcription factor with high similarity to TCP14 proteins from *A. thaliana* and *Gossypium raimondii* (figs. S3 and S4), as the dominant interactor of AaORA (9 of 23 positive clones). The physical interaction between AaORA and AaTCP14 was further confirmed by both Y2H (Fig. 1A) and in vitro GST pulldown assays with proteins purified from *Escherichia coli* (Fig. 1B).

BiFC assays were performed to verify the AaORA-AaTCP14 interaction in plant cells. When AaORA-cYFP (the C-terminal fragment of YFP) was transiently coexpressed with AaTCP14-nYFP (the N-terminal fragment of YFP) in *Nicotiana benthamiana* leaf cells, reconstituted YFP fluorescence was observed in the nucleus. By contrast, no YFP fluorescence signals were observed when AaORA-cYFP and nYFP or cYFP and AaTCP14-nYFP were coexpressed (Fig. 1C). These results suggest that AaORA interacts with AaTCP14 in planta. Coimmunoprecipitation (Co-IP) assays further demonstrated that AaORA associates with AaTCP14 in *N. benthamiana* leaf cells (Fig. 1D). Together, these results demonstrate that AaORA directly interacts with AaTCP14 in vitro and in vivo.

### *AaTCP14* and *AaORA* have similar expression patterns and AaTCP14 is a nuclear-localized protein

**Fig. 2:**
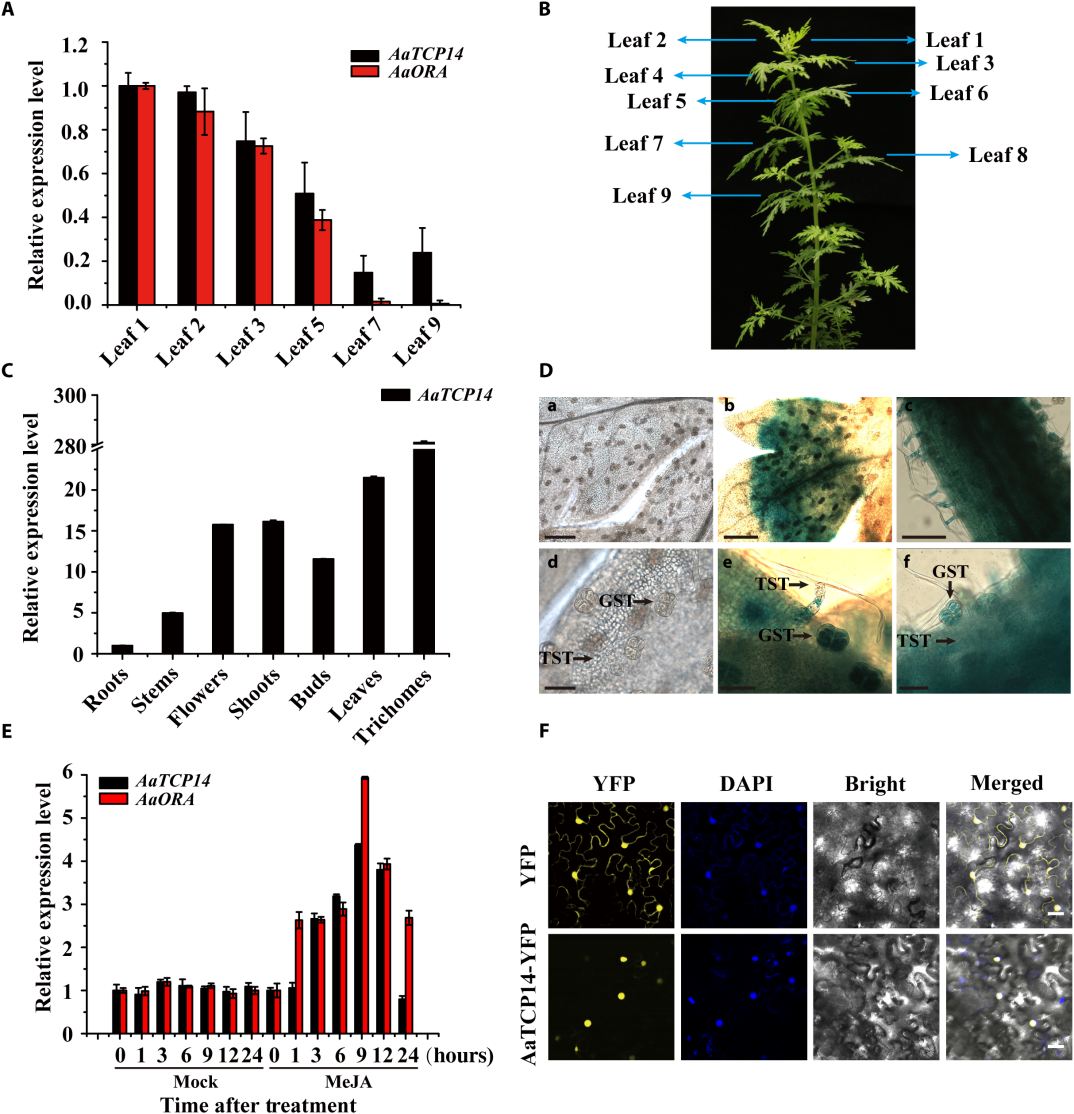
Expression pattern and subcellular localization of AaTCP14. (**A**) Relative expression levels of *AaTCP14* and *AaORA* in leaves at different positions. The expression levels of *AaTCP14* and *AaORA* in leaf 1 were set as 1. *Actin* was used as an internal control. The data represent the means ± SD of three replicates from three independent *A. annua* plants. (**B**) An image of a 3-month-old *A. annua* plant labeled with the leaves numbered as in (A). (**C**) Relative expression levels of *AaTCP14* in roots, stems, flowers, shoots, buds, leaves, and trichomes were measured by qRT-PCR. The expression level of *AaTCP14* in roots was set as 1. *Actin* was used as an internal control. The data represent the means ± SD of three replicates from three independent *A. annua* plants. (**D**) GUS expression (blue staining) in *A. annua* plants transformed with the *1391Z-GUS* empty vector (control plants) and *1391Z*-*proTCP14*-*GUS*. (**a** and **d**) Leaves of control plants. (**b** and **e**) Leaves of *1391Z*-*proTCP14*-*GUS* plants. (**c** and **f**) Stems of *1391Z*-*proTCP14*-*GUS* plants. Scale bars, 200 μm (a to c) and 50 μm (d to f). (**E**) Relative expression levels of *AaTCP14* and *AaORA* in plants treated with MeJA (100 μM) over 24 hours. *Actin* was used as an internal control. The data represent the means ± SD of three replicates from three independent experiments. (**F**) Subcellular localization of AaTCP14. Colocalization of AaTCP14-YFP and nuclei was determined by DAPI staining. YFP was used as a negative control. Three independent transfection experiments were performed. Scale bars, 20 μm.

To investigate the spatial and temporal expression pattern of *AaTCP14*, we analyzed the expression levels of *AaTCP14* in leaves at different positions and in different tissues using quantitative real-time polymerase chain reaction (qRT-PCR). *AaTCP14* expression was high in young leaves (leaf 1 and leaf 2) and then gradually decreased during leaf development (Fig. 2, A and B). This expression pattern is similar to that of artemisinin biosynthetic genes (*22*) and *AaORA* (Fig. 2, A and B). In addition, *AaTCP14* transcripts were also detected in other organs. *AaTCP14* was most highly expressed in trichomes; moderately expressed in leaves, shoots, flowers, and buds; lowly expressed in the stems; and very lowly expressed in the roots (Fig. 2C).

To more precisely determine the expression patterns of *AaTCP14*, we transformed wild-type (WT) *A. annua* with *1391Z*-*proTCP14-GUS* (β-glucuronidase), where expression of the GUS reporter is driven by the 1828–base pair (bp) promoter sequence of *AaTCP14*. Histochemical GUS staining of *1391Z*-*proTCP14-GUS* transgenic lines revealed GUS activity in young leaves and stems, especially in the two types of trichomes, T-shaped non-GSTs (TSTs) and GSTs (Fig. 2D). No GUS activity was observed in *A. annua* plants transformed with the empty vector (Fig. 2D). The expression pattern of the GUS reporter correlated well with the expression patterns determined using qRT-PCR (Fig. 2C).

There is accumulating evidence that the phytohormone MeJA plays a positive role in artemisinin biosynthesis (*37*). *AaORA* expression was gradually induced by MeJA and peaked at 9 hours after MeJA treatment (Fig. 2E). Thus, we wanted to know whether *AaTCP14* had the same expression pattern as *AaORA* under MeJA treatment. On the basis of qRT-PCR analysis, *AaTCP14* expression was robustly induced at 3 hours and also peaked at 9 hours after MeJA treatment (Fig. 2E). Thus, these results suggest that *AaTCP14* and *AaORA* have similar expression patterns in response to MeJA treatment, which also induces artemisinin biosynthesis.

To determine where the AaTCP14 protein functions within the cell, we next determined the subcellular localization of an AaTCP14-YFP fusion protein. In contrast to YFP, which was distributed throughout the cell, the AaTCP14-YFP fusion protein was observed exclusively in nuclei (Fig. 2F) in *N. benthamiana* leaf cells, suggesting that AaTCP14 is a nuclear-localized protein, consistent with its potential function as a transcriptional regulator.

### Overexpression of *AaTCP14* increases artemisinin content, and attenuated expression of *AaTCP14* reduces artemisinin production in *A. annua*

**Fig. 3:**
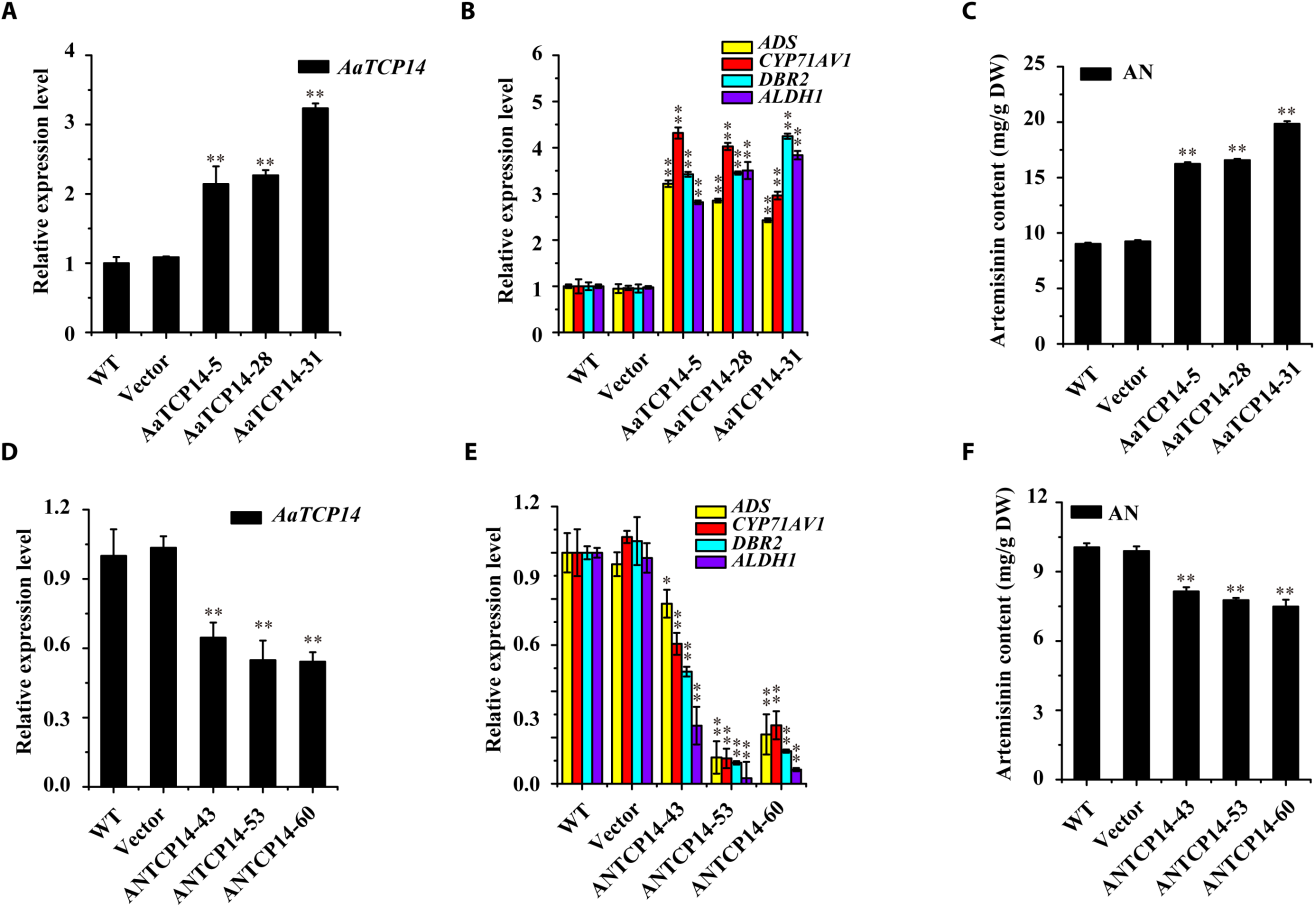
Analysis of *AaTCP14* transgenic plants. (**A** and **D**) Expression levels of *AaTCP14* in different *A. annua AaTCP14* overexpression (A) and antisense lines (D), plants transformed with the empty vector, and WT plants. *Actin* was used as the internal standard. (**B** and **E**) Expression levels of *ADS*, *CYP71AV1*, *DBR2*, and *ALDH1* in different *A. annua AaTCP14* overexpression (B) and antisense lines (E), plants transformed with the empty vector, and WT. *Actin* was used as the internal control. (**C** and **F**) HPLC analysis of artemisinin (AN) in the leaves of different *A. annua AaTCP14* overexpression (C) and antisense lines (F), plants transformed with the empty vector, and WT. All data represent the means ± SD of three replicates from three cutting propagations. *P < 0.05, **P < 0.01, Student’s *t* test.

To investigate the physiological role of AaTCP14, *AaTCP14* was overexpressed in *A. annua* plants. Three independent *AaTCP14* overexpression lines, named AaTCP14-5, AaTCP14-28, and AaTCP14-31, were selected for subsequent analysis. The transcript levels of *AaTCP14* in these lines were significantly higher than those in WT plants (Fig. 3A). The expression levels of *ADS*, *CYP71AV1*, *DBR2*, and *ALDH1* were also dramatically increased in *AaTCP14* overexpression plants compared with WT (Fig. 3B). This suggests that AaTCP14 may activate the transcription of artemisinin biosynthetic genes. Furthermore, the expression levels of *AaWRKY1*, *AaMYC2*, *AaGSW1*, and *AaORA*, which are involved in different pathways positively regulating artemisinin biosynthesis (*16*–*18*, *22*), were significantly up-regulated in the *AaTCP14* overexpression lines (fig. S5, A to D). Up-regulation of these genes may, in turn, activate *ADS*, *CYP71AV1*, and *DBR2* expression. In addition, the expression levels of some well-known JA biosynthetic genes, including *AaAOC* and *AaOPR3*, were higher in *AaTCP14* overexpression lines than in WT, but the expression levels of *AaAOS* and *AaOPCL1* were not (fig. S5, E to H). Up-regulation of *AaAOC* could enhance the JA content, which, in turn, would activate artemisinin biosynthesis (*38*). Together, these results indicate that AaTCP14 has the potential to enhance artemisinin yield. Consistent with the up-regulation of genes involved in artemisinin biosynthesis, high-performance liquid chromatography (HPLC) analysis showed that the artemisinin and DHAA contents in *AaTCP14* overexpression lines increased by 80 to 120% and 10 to 120%, respectively, compared with WT (Fig. 3C and fig. S6A), whereas the AA content decreased by 45 to 69% in *AaTCP14* overexpression plants (fig. S6B). These results indicate that AaTCP14 positively promotes artemisinin biosynthesis.

To further verify the biological role of *AaTCP14* in controlling artemisinin biosynthesis, we silenced *AaTCP14* expression by transforming *A. annua* with the antisense vector *pHB-ANTCP14*. Three candidate *AaTCP14* antisense transgenic plants, named ANTCP14-43, ANTCP14-53, and ANTCP14-60, were chosen for subsequent analysis. The expression level of *AaTCP14* in these antisense lines was significantly reduced compared with WT (Fig. 3D), and the expression levels of *ADS*, *CYP71AV1*, *DBR2*, and *ALDH1* were significantly down-regulated compared with WT (Fig. 3E). The expression levels of *AaWRKY1*, *AaMYC2*, *AaGSW1*, and *AaORA* were also down-regulated in the *AaTCP14* antisense plants (fig. S5, I to L), which probably contributed to decreased *ADS*, *CYP71AV1*, and *DBR2* expression. Moreover, the expression levels of *AaAOC* and *AaOPR3* decreased in *AaTCP14* antisense plants, but the expression levels of *AaAOS* and *AaOPCL1* did not (fig. S5, M to P). This is consistent with the gene expression changes observed in the *AaTCP14* overexpression plants and suggests that AaTCP14 regulates JA biosynthesis through *AaAOC* and *AaOPR3*. Together, these results indicate that AaTCP14 plays a positive role in controlling artemisinin biosynthesis. Consistent with this, the contents of artemisinin, DHAA, and AA in *AaTCP14* antisense plants were reduced by 19 to 25%, 20 to 72%, and 11 to 37%, respectively (Fig. 3F and fig. S6, D and E). No obvious morphological differences were observed in either the *AaTCP14* overexpression or antisense transgenic plants compared with the *A. annua* plants transformed with the empty vector (control plants, labeled as vector) (fig. S6, C and F). Together, these results indicate that AaTCP14 is a positive regulator of artemisinin biosynthesis and may be a good target in efforts to increase artemisinin production through genetic engineering of *A. annua*.

### AaTCP14 enhances the transcription of both *DBR2* and *ALDH1* by binding to their promoters

**Fig. 4:**
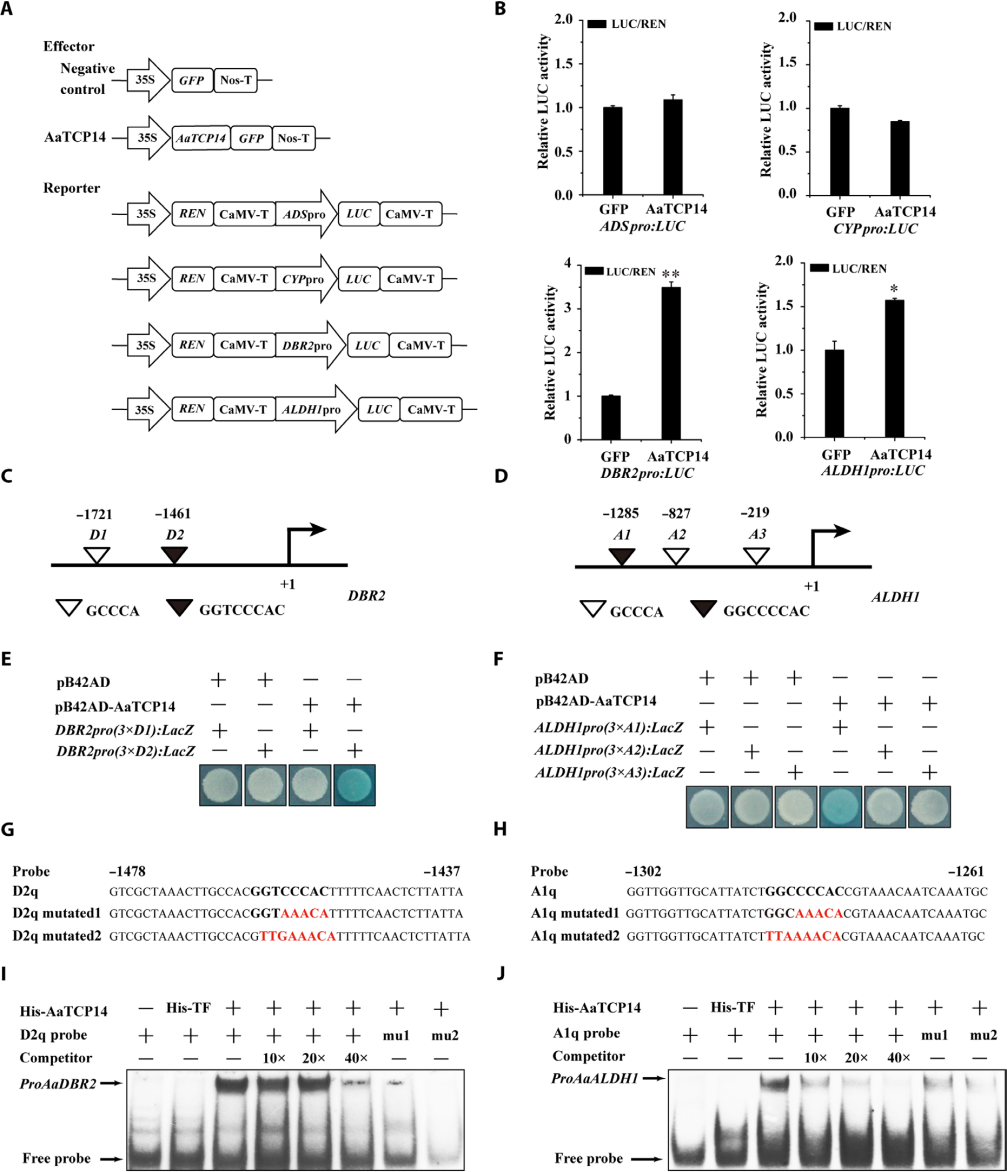
AaTCP14 is a transcriptional activator of *DBR2* and *ALDH1*. (**A**) Schematic diagrams of the effector (pCambia1300-AaTCP14-GFP) and reporter (35S: REN-ADS/CYP/DBR2/ALDH1_pro_:LUC) plasmids used in dual-LUC assays. CYP, CYP71AV1; REN, *Renilla* luciferase; LUC, firefly luciferase. (**B**) Dual-LUC assay in *N. benthamiana* cells using the constructs shown in (A). The GFP effector was used as a negative control, and the LUC/REN ratios of GFP were set as 1. Three independent transfection experiments were performed. The data represent the means ± SD of three replicates from three independent experiments. *P < 0.05, **P < 0.01, Student’s *t* test. (**C** and **D**) Schematic diagrams of the *DBR2* and *ALDH1* promoters. The positions of potential TBS DNA binding sites (*D1* and *D2* and *A1*, *A2*, and *A3*) are shown as black and white triangles and are numbered on the basis of their distance from the translational start site (ATG), which is set as +1. (**E** and **F**) Y1H assays showing that AaTCP14 binds to the TBS motifs of *DBR2* and *ALDH1*. Three tandem repeats of each motif were used as baits. Yeast cells coexpressing pB42AD, pB42AD-AaTCP14, and the DNA motifs from the *DBR2* and *ALDH1* promoters were grown on selective medium, SD/-Trp/-Ura, containing X-gal (20 mg/liter), and pictures were taken after 4 days of incubation at 30°C. Blue plaques indicate protein-DNA interactions. The Y1H assays were repeated three times, and representative results are shown. (**G** and **H**) The sequences of WT and mutated probes used for EMSAs. Class I TCP binding motifs are shown in bold, and the mutated nucleotides are indicated in red. (**I** and **J**) EMSAs showing that AaTCP14 binds to the *D2q* motif from *DBR2* and the *A1q* motif from *ALDH1*. Unlabeled D2q and A1q were used as cold competitors, and two labeled mutated D2q and A1q probes were tested as negative controls. 10×, 20×, and 40× indicate the fold excess of cold competitors relative to that of the labeled probe. His-TF protein was used as a negative control.

To further investigate whether AaTCP14 directly affects *ADS*, *CYP71AV1*, *DBR2*, and *ALDH1* expression, we used dual-LUC assays. When AaTCP14-GFP (green fluorescent protein) was expressed in *N. benthamiana* leaf cells harboring the DBR2_pro_:LUC or ALDH1_pro_:LUC plasmids, the promoter activities of *DBR2* and *ALDH1*, respectively, significantly increased compared with the GFP control (Fig. 4, A and B). However, the activities of the *ADS* and *CYP71AV1* promoters were not obviously altered (Fig. 4B). Together, these results suggest that AaTCP14 is a positive regulator of *DBR2* and *ALDH1*.

Previous studies suggested that TCP family proteins directly bind to the TCP binding site (TBS) in the promoter sequence of target genes in *A. thaliana* (*27*, *28*, *33*). Promoter analysis revealed two similar TBS motifs (*D1* and *D2*; 1721 and 1461 bp upstream of ATG, respectively) in the promoter of *DBR2* and three candidate TBS motifs (*A1*, *A2*, and *A3*; 1285, 827, and 219 bp upstream of ATG, respectively) in the *ALDH1* promoter (Fig. 4, C and D). Y1H assays showed that binding of the pB42AD-AaTCP14 fusion protein, but not pB42AD alone, to three tandem repeats of the *D2* motif or the *A1* motif strongly activated the expression of the *LacZ* reporter gene (Fig. 4, E and F), indicating that AaTCP14 binds to the *D2* and *A1* motifs in the *DBR2* and *ALDH1* promoters, respectively.

Next, to further confirm AaTCP14 binding to *D2* and *A1*, electrophoretic mobility shift assays (EMSAs) were conducted with His-AaTCP14 and His-TF (trigger factor) proteins purified from *E. coli*. As shown in Fig. 4 (I and J), a single shifted band was observed in the presence of both His-AaTCP14 and a labeled DNA probe containing the *D2* or *A1* motif (D2q or A1q, shown in Fig. 4, G and H). The intensity of this band decreased with increasing concentrations of a cold competitor, and no band was observed when His-TF was added in place of His-AaTCP14 (Fig. 4, I and J). Moreover, mutations in the *D2* or *A1* motifs markedly attenuated the band intensity (Fig. 4, G to J), indicating that AaTCP14 specifically bound to the *D2q* and *A1q* motifs. Together, these results demonstrate that AaTCP14 positively regulates *DBR2* and *ALDH1* expression by directly binding to their promoters.

### AaTCP14 and AaORA synergistically promote artemisinin biosynthesis, and the function of AaORA is partly dependent on AaTCP14

**Fig. 5:**
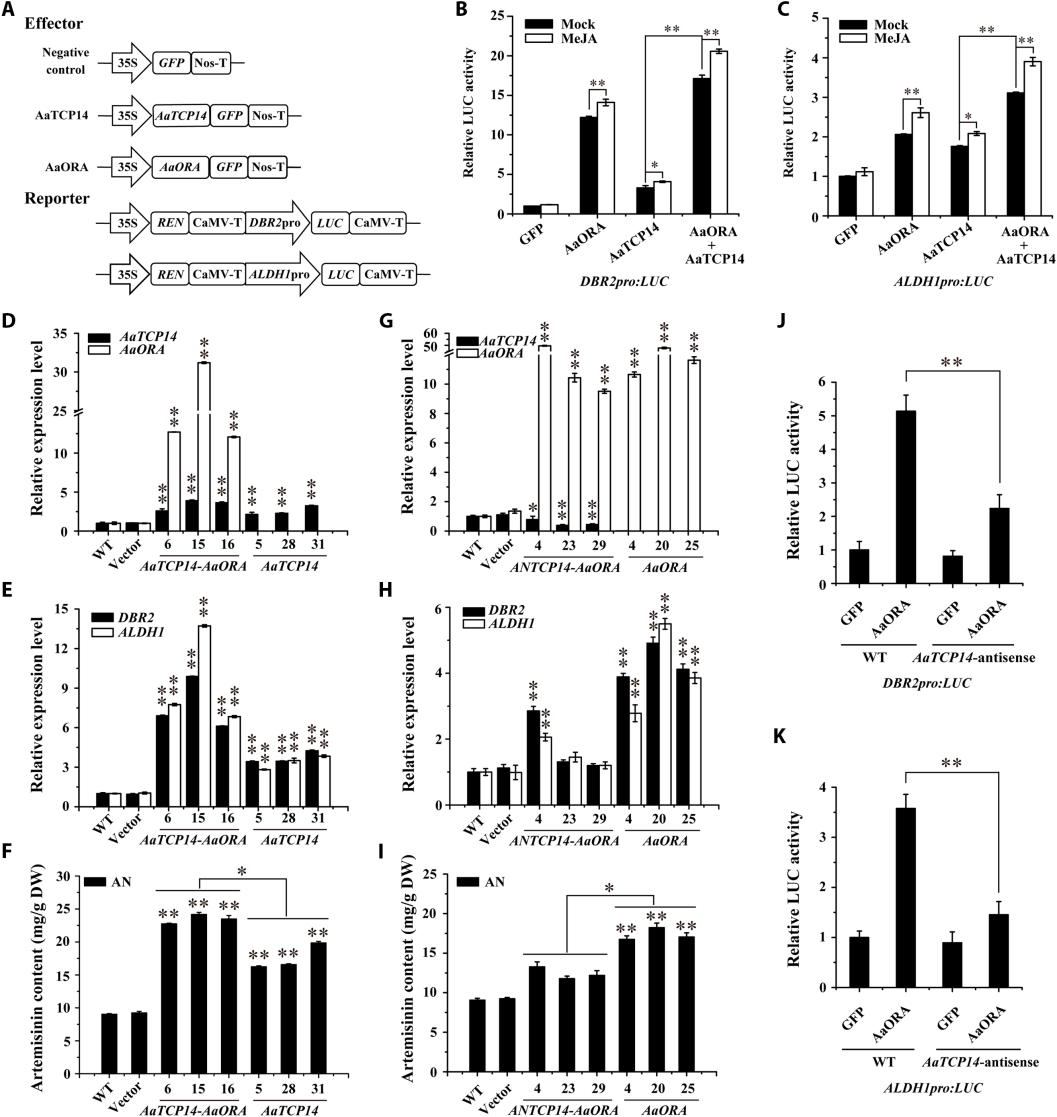
AaTCP14 and AaORA synergistically promote artemisinin biosynthesis, and AaORA is partly dependent on AaTCP14. (**A**) A schematic representation of the constructs used in dual-LUC assays. (**B** and **C**) Activation of the *DBR2* (B) and *ALDH1* (C) promoters by AaORA and AaTCP14 proteins in the presence or absence of MeJA in *N. benthamiana* leaves. The GFP effector in the mock treatment served as a negative control, and the LUC/REN ratios of GFP were set as 1. Three independent transfection experiments were performed. The reporter strain harboring *DBR2pro:LUC* or *ALDH1pro:LUC* was mixed with the effector strains harboring *35Spro:AaTCP14* and *35Spro:AaORA* at a ratio of 1:1:1. The data represent the means ± SD of three replicates from three independent experiments. *P < 0.05, **P < 0.01, Student’s *t* test. (**D** and **E**) Expression levels of *AaTCP14* and *AaORA* (D) and *DBR2* and *ALDH1* (E) in different *A. annua* plants including *AaTCP14-AaORA* co-overexpression (*AaTCP14-AaORA*), *AaTCP14* overexpression lines, and plants transformed with the empty vector. *Actin* was used as the internal standard. WT plants served as controls. The data represent the means ± SD of three replicates from three cutting propagations. **P < 0.01, Student’s *t* test. (**F**) HPLC analysis of artemisinin (AN) in the leaves of different *A. annua* plants including *AaTCP14-AaORA* and *AaTCP14* overexpression lines, plants transformed with the empty vector, and the WT control. The data represent the means ± SD of three replicates from three cutting propagations. *P < 0.05, **P < 0.01, Student’s *t* test. (**G** and **H**) Expression levels of *AaTCP14* and *AaORA* (G) and *DBR2* and *ALDH1* (H) in different *A. annua* plants including the *AaTCP14* antisense–*AaORA* overexpression (*ANTCP14*-*AaORA*), *AaORA* overexpression lines, and plants transformed with the empty vector. *Actin* was used as the internal standard. WT plants served as controls. The data represent the means ± SD from three replicates from three cutting propagations. *P < 0.05, **P < 0.01, Student’s *t* test. (**I**) HPLC analysis of artemisinin (AN) in the leaves of different *A. annua* plants including the *ANTCP14*-*AaORA*, *AaORA* overexpression lines, plants transformed with the empty vector, and the WT control. The data represent the means ± SD of three replicates from three cutting propagations. *P < 0.05, **P < 0.01, Student’s *t* test. (**J** and **K**) Dual-LUC experiments showing the activation of the *DBR2* (J) and *ALDH1* (K) promoters by AaORA in *A. annua* protoplasts from WT and *AaTCP14* antisense (*ANTCP14*) lines. GFP was used as a negative control, and the LUC/REN ratios of GFP in WT *A. annua* were set as 1. Three independent transfection experiments were performed. The data represent the means ± SD of three independent experiments. Student’s *t* test, **P < 0.01.

We have shown that the AaTCP14 and AaORA proteins interact (Fig. 1), that the genes encoding these proteins share similar expression patterns (i.e., in leaves sampled from different positions and in response to MeJA treatment) (Fig. 2, A and E), and that both proteins activate the *DBR2* and *ALDH1* promoters (Fig. 4 and fig. S1B). These results prompted us to examine whether AaORA affects the AaTCP14-mediated transactivation of *DBR2* and *ALDH1* under MeJA treatment. To test this, we used dual-LUC assays to evaluate the promoter activity of *DBR2* and *ALDH1* with the effectors AaTCP14 and AaORA delivered individually or in combination in the presence or absence of MeJA in *N. benthamiana* leaf cells. AaTCP14 and AaORA alone were able to significantly activate the *DBR2* and *ALDH1* promoters, and this induction was significantly enhanced by coexpressing AaORA and AaTCP14 (Fig. 5, A to C). These results suggest that AaORA elevates the transcriptional activation activity of AaTCP14 through direct protein interactions (Fig. 1). It should be noted that MeJA treatment strengthened the activation of the *DBR2* and *ALDH1* promoters by AaTCP14, AaORA, and AaTCP14-AaORA (Fig. 5, B and C), suggesting that AaTCP14 and AaORA may act as downstream components of JA signaling pathway and cooperatively activate the expression of *DBR2* and *ALDH1*.

Next, to check whether the AaORA-AaTCP14 interaction affects the ability of AaTCP14 to bind to the *DBR2* and *ALDH1* promoters, we performed EMSAs using DNA fragments D2q and A1q (labeled in Fig. 4, G and H), respectively, as probes. The addition of an increasing amount of His-AaORA fusion protein, which was purified from *E. coli*, had a negligible effect on the ability of His-AaTCP14 to bind to *DBR2* and *ALDH1* (fig. S7, A and B), indicating that AaORA does not affect the ability of AaTCP14 to bind to these promoters in vitro. Together, these observations support the notion that the physical interaction between AaORA and AaTCP14 may promote the transcriptional activation activity of AaTCP14 but does not affect its DNA binding ability.

To evaluate the importance of the AaTCP14-AaORA complex in artemisinin biosynthesis, we generated transgenic *A. annua* plants simultaneously overexpressing *AaTCP14* and *AaORA*. Three candidate *AaTCP14*-*AaORA* co-overexpression lines, named AaTCP14–AaORA-6, AaTCP14–AaORA-15, and AaTCP14–AaORA-16, were chosen for further analysis. The expression levels of *AaTCP14* and *AaORA* in these lines were significantly higher (2.5- to 4.0-fold and 12- to 31-fold, respectively) than those in WT (Fig. 5D). The expression levels of *DBR2* and *ALDH1* dramatically increased in *AaTCP14*-*AaORA* coexpression plants compared with both WT and plants overexpressing only *AaTCP14* (Fig. 5E), indicating that AaTCP14 and AaORA cooperatively activate the transcription of *DBR2* and *ALDH1*. This finding is consistent with the enhancement of *DBR2* and *ALDH1* promoter activation by coexpressing *AaTCP14* and *AaORA* in *N. benthamiana* leaf cells (Fig. 5, A to C). Furthermore, HPLC analysis showed that the levels of artemisinin, DHAA, and AA were significantly increased in *AaTCP14*-*AaORA* co-overexpression plants compared with WT and plants overexpressing only *AaTCP14* (Fig. 5F and fig. S6, G and H). No obvious morphological differences were observed between *AaTCP14*-*AaORA* coexpression plants and *A. annua* plants transformed with the empty vector (fig. S6I). Together, these results indicate that AaTCP14 and AaORA synergistically regulate artemisinin biosynthesis and may form a protein complex that enhances artemisinin production.

To determine whether the function of AaORA is dependent on AaTCP14, we generated transgenic *A. annua* plants only overexpressing *AaORA* and plants simultaneously overexpressing *AaORA* and silenced *AaTCP14*. Three candidate *AaORA* overexpression lines, AaORA-4, AaORA-20, and AaORA-25, and three *AaTCP14* antisense–*AaORA* overexpression lines, ANTCP14–AaORA-4, ANTCP14–AaORA-23, and ANTCP14–AaORA-29, were chosen for further analysis. The expression levels of *AaORA* in the *AaORA* overexpression and *AaTCP14* antisense–*AaORA* overexpression plants were notably higher than those in WT (Fig. 5G). The expression levels of *AaTCP14* in the *AaTCP14* antisense–*AaORA* overexpression plants were markedly lower than those in WT (Fig. 5G). The expression levels of *DBR2* and *ALDH1* in the *AaTCP14* antisense–*AaORA* overexpression plants were higher than those in WT but lower than those in *AaORA* overexpression plants (Fig. 5H). Consistently, the artemisinin content in the *AaTCP14* antisense–*AaORA* overexpression plants were higher than those in WT but lower than those in *AaORA* overexpression plants (Fig. 5I). Dual-LUC assays were also performed to evaluate the regulation of the *DBR2* and *ALDH1* promoters by AaORA in protoplasts from WT and *ANTCP14 A. annua* lines. The transactivation of the *DBR2* and *ALDH1* promoters by AaORA was much lower in cells with less *AaTCP14* expression than in WT cells (Fig. 5, J and K). Collectively, these results demonstrate that the enhancement of artemisinin production by AaORA is partly dependent on AaTCP14.

### AaJAZ8 negatively regulates artemisinin biosynthesis and interacts with both AaTCP14 and AaORA

It has been reported that JA induces artemisinin accumulation by activating artemisinin biosynthetic genes or JA-responsive transcription factors (*17*, *18*, *37*). In contrast, JAZ proteins function as negative regulators that repress diverse JA responses by suppressing various positive transcriptional regulators (*42*–*44*). In *A. annua*, the JAZ protein encoded by *AaJAZ8* was previously found to repress the transcriptional activation activity of AaHD1, which is involved in trichome development and artemisinin content (*42*). In our study, we found that *AaJAZ8* is broadly expressed and is most highly expressed in trichomes (fig. S8A). Moreover, *AaJAZ8* expression is induced by MeJA treatment, and the level of AaJAZ8 protein is regulated by MeJA through the 26S proteasome (fig. S8, B and C), suggesting the potential role of AaJAZ8 in controlling JA-mediated artemisinin accumulation. To functionally characterize AaJAZ8 and determine its role in artemisinin biosynthesis, we generated transgenic plants overexpressing *AaJAZ8*. The expression levels of *ADS*, *CYP71AV1*, *DBR2*, and *ALDH1*, determined by qRT-PCR analysis, were lower in *AaJAZ8* overexpression lines than in WT plants (fig. S8D), and the artemisinin content was concomitantly decreased (fig. S8E). Because the jas domain of JAZ proteins is essential for JA-mediated protein degradation, we also overexpressed a dominant form of *AaJAZ8* (*AaJAZ8*Δ*jas*) that lacks the jas domain to disrupt JA-mediated AaJAZ8 protein degradation. The expression levels of *ADS*, *CYP71AV1*, *DBR2*, and *ALDH1* were lower in *AaJAZ8*Δ*jas* overexpression lines than in WT plants (fig. S8F). Together, these results suggest that AaJAZ8 attenuates JA-induced artemisinin biosynthesis in *A. annua*.

**Fig. 6:**
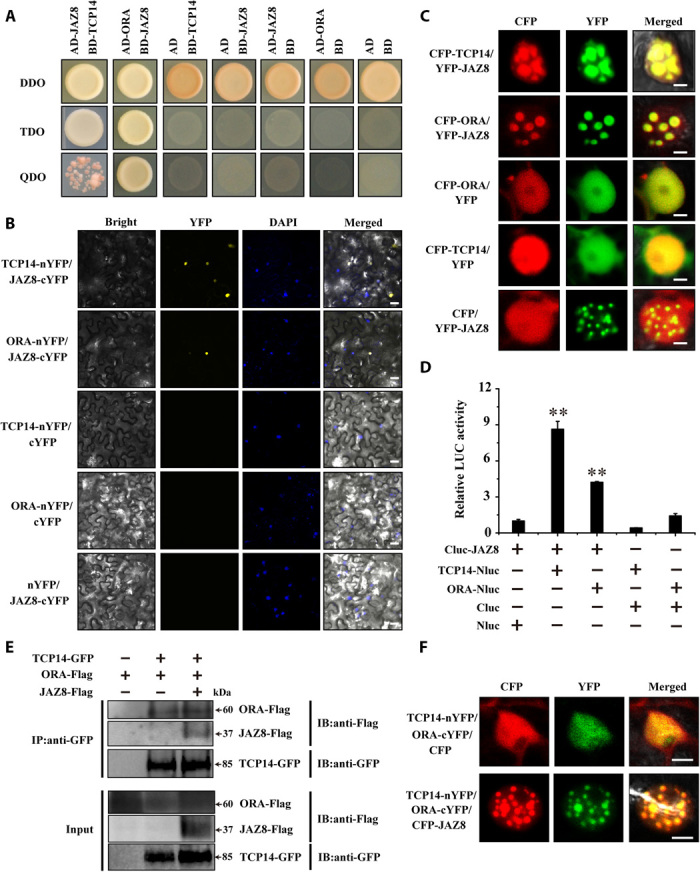
AaJAZ8 interacts with both AaTCP14 and AaORA. (**A**) Y2H assays to detect the pairwise interactions between AaJAZ8, AaTCP14, and AaORA. AaORA and AaJAZ8 were fused with the activation domain (AD), and AaTCP14 and AaJAZ8 were fused with the binding domain (BD). Yeast cells harboring bait and prey plasmids were grown on two types of selective media, QDO and TDO, and the control medium, DDO. Pictures were taken after 4 days of incubation at 30°C. The Y2H assays were repeated three times, and representative results are shown. (**B**) BiFC analysis to detect the pairwise interactions between AaTCP14, AaORA, and AaJAZ8. AaTCP14 and AaORA were fused to the N-terminal fragment of YFP (TCP14-nYFP and ORA-nYFP), and AaJAZ8 was fused to the C-terminal fragment of YFP (JAZ8-cYFP). Colocalization of reconstituted YFP and nuclei was determined by DAPI staining. Three independent transfection experiments were performed. Scale bars, 20 μm. (**C**) Colocalization of AaTCP14 and AaJAZ8 and of AaORA and AaJAZ8 to the same NBs in *N. benthamiana* cells. *N. benthamiana* leaves were infiltrated with *A. tumefaciens* strains harboring different combinations of CFP-TCP14, CFP-ORA, and YFP-JAZ8 fusion protein constructs, and CFP-TCP14 and YFP, CFP-ORA and YFP, and CFP and YFP-JAZ8 were cotransformed as negative controls. Pictures were taken after 60 to 72 hours of incubation at 23°C. Three independent transfection experiments were performed. Scale bars, 5 μm. (**D**) LUC complementation assay to detect the pairwise interactions between AaTCP14, AaORA, and AaJAZ8. AaJAZ8 was fused to the C-terminal fragment of LUC (Cluc-JAZ8), and AaTCP14 and AaORA were fused to the N-terminal fragment of LUC (TCP14-Nluc and ORA-Nluc). LUC activity of Cluc-JAZ8 and Nluc was set to 1. Three independent transfection experiments were performed. The data represent the means ± SD of three independent experiments. **P < 0.01, Student’s *t* test. (**E**) Co-IP analysis of TCP14-GFP, ORA-Flag, and JAZ8-Flag in *N. benthamiana* leaves. Total protein extracts from *N. benthamiana* leaves infiltrated with *A. tumefaciens* harboring TCP14-GFP, ORA-Flag, and JAZ8-Flag fusion protein constructs were immunoprecipitated with anti-GFP antibody. The coimmunoprecipitated proteins were detected by anti-Flag antibody. Similar results were obtained in three independent experiments. (**F**) Colocalization of TCP14-nYFP, ORA-cYFP, and CFP-JAZ8 in the same NBs in *N. benthamiana* leaves. Colocalization of TCP14-nYFP, ORA-cYFP, and CFP was used as the negative control. After *A. tumefaciens* infiltration, pictures were taken after 60 to 72 hours of incubation at 23°C. Three independent transfection experiments were performed. Scale bars, 5 μm. IB, immunoblotting.

Considering that *AaTCP14*, *AaORA*, and *AaJAZ8* are all induced by MeJA (Fig. 2E and fig. S8B) and that AaORA is a putative downstream regulator of JA signaling (*17*), there may be a possible connection between *AaTCP14*, *AaORA*, and *AaJAZ8*. Hence, we examined whether AaJAZ8 could physically interact with AaTCP14 or AaORA. Y2H assays revealed that both AaTCP14 and AaORA interact with AaJAZ8 (Fig. 6A). Intriguingly, among all the AaJAZ proteins (AaJAZ1 to AaJAZ9), only AaJAZ8 interacted with AaTCP14, and apart from AaJAZ7, all AaJAZ proteins (AaJAZ1 to AaJAZ6, AaJAZ8, and AaJAZ9) interacted with AaORA (fig. S9A), indicating that AaTCP14 and AaORA are common targets of AaJAZ8. MYC2 is a key regulator of JA signaling and is also a general target of JAZ proteins in *Arabidopsis* (*43*). AaMYC2 and AaJAZ1 to AaJAZ4, which were previously reported to interact, were used as positive controls (*18*), and parallel experiments showed clear interactions of AaMYC2 with all other AaJAZ proteins (AaJAZ5, AaJAZ6, AaJAZ8, and AaJAZ9) except AaJAZ7 (fig. S9A). Next, the pairwise interactions of AaJAZ8 with AaTCP14 and AaORA were validated by performing BiFC assays. As expected, when the AaJAZ8-cYFP construct was transiently coexpressed with AaTCP14-nYFP or AaORA-nYFP in *N. benthamiana* leaf cells, strong YFP fluorescence signals were observed in the nucleus (Fig. 6B), indicating that AaJAZ8 interacts with AaTCP14 and AaORA in plant cells.

To further substantiate this result, we performed transient colocalization assays, expressing YFP-AaJAZ8, cyan fluorescent protein (CFP)–AaTCP14, and CFP-AaORA, either individually or together, in *N. benthamiana* leaf cells. As anticipated, AaJAZ8 colocalized with AaTCP14 and with AaORA in the same nuclear bodies (NBs) (Fig. 6C). AaJAZ8-AaTCP14 and AaJAZ8-AaORA interactions in *N. benthamiana* leaf cells were also confirmed by LUC complementation assays (Fig. 6D). Together, these results suggest that AaJAZ8 specifically interacts with AaTCP14 and AaORA in plant cells. Next, Y2H assays were carried out to map which domain of AaJAZ8 is responsible for its interaction with AaTCP14 and AaORA. AaJAZ8Δjas, which contains only the ZIM domain, was capable of interacting with AaTCP14 and AaORA, but truncated versions of AaJAZ8 (AaJAZ8ΔZIM) that lacked the ZIM domain could not (fig. S9B).

Subsequently, Co-IP assays were performed to examine the interactions between all three proteins. When AaORA-Flag and AaJAZ8-Flag were coexpressed with AaTCP14-GFP in *N. benthamiana* leaf cells, both AaORA and AaJAZ8 were coimmunoprecipitated with AaTCP14 (Fig. 6E). To further explore the interaction between AaJAZ8 and the AaTCP14-AaORA complex, we transiently coexpressed AaTCP14-nYFP and AaORA-cYFP with CFP, and strong YFP fluorescent signals corresponding to reconstituted YFP were observed uniformly throughout the nucleus (Fig. 6F). However, when AaTCP14-nYFP and AaORA-cYFP were coexpressed with AaJAZ8-CFP, we found that the reconstituted YFP colocalized with AaJAZ8 in the same NBs (Fig. 6F) in *N. benthamiana* leaf cells, suggesting that AaJAZ8 brings the AaTCP14-AaORA complex to the NBs.

To gain more insight into the interactions between AaTCP14, AaJAZ8, and AaORA, we mapped the domains of AaORA that interact with AaTCP14 and AaJAZ8. We found that AaORAΔN1, which contains only the C terminus of AaORA, was capable of interacting with AaTCP14, but truncated versions of AaORA (AaORAΔC1, AaORAΔC2, and AaORAMC) that lacked the C-terminal domain could not (fig. S9C). However, the N-terminal or C-terminal domain of AaORA alone was sufficient for interacting with full-length AaJAZ8 (fig. S9C). These results suggest that AaORA uses different domains for the AaTCP14-AaORA and AaJAZ8-AaORA interactions.

### AaJAZ8 attenuates the binding between AaTCP14 and AaORA

**Fig. 7:**
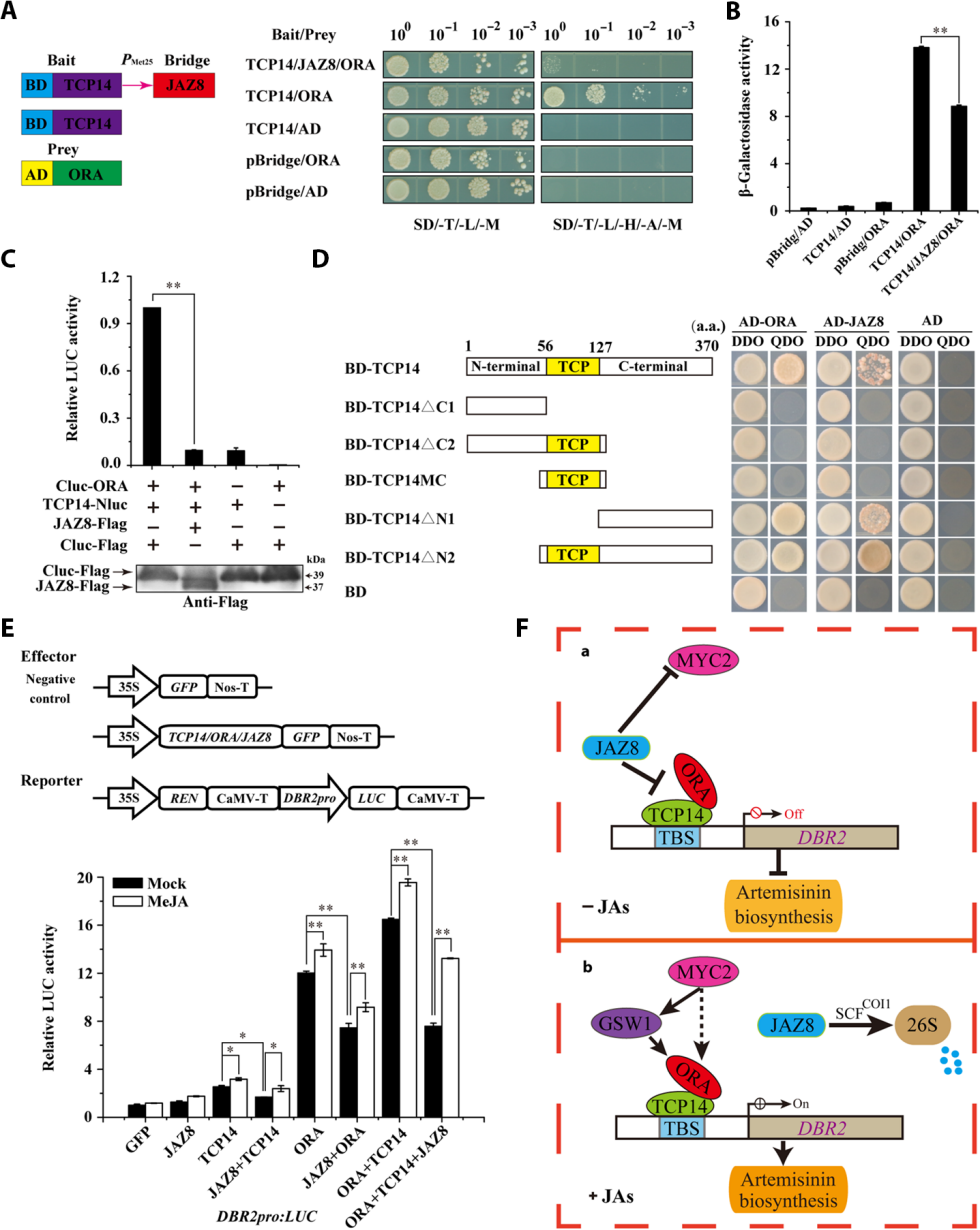
AaJAZ8 interferes with the interaction between AaORA and AaTCP14 and attenuates the transcriptional activation activity of AaTCP14 and AaORA. (**A**) Y3H assays of the influence of JAZ8 on TCP14-ORA interaction. Left, schematic representation of the bait and prey constructs used in Y3H assays. ORA was fused with the activation domain (AD), and TCP14 was fused with the binding domain (BD). *P*_Met25_ is an inducible promoter that drives the expression of the bridge protein, JAZ8. Right, yeast cells harboring bait and prey plasmids were grown on two types of selective media, SD/-Trp/-Leu/-Met (SD/-T/-L/-M) and SD/-Trp/-Leu/-His/-Ade/-Met (SD/-T/-L/-H/-A/-M), and pictures were taken after 4 days of incubation at 30°C. The different dilutions, 10^0^, 10^−1^, 10^−2^, and 10^−3^, are shown at the top of the figure. The Y3H assays were repeated three times, and representative results are shown. (**B**) β-Gal activities of yeast in (A) were measured in the presence or absence of JAZ8. The promoter driving *JAZ8* expression was suppressed by methionine. The data represent the means ± SD of three replicates from three independent experiments. Student’s *t* test, **P < 0.01. The β-gal activity assays were repeated three times, and similar results were obtained. (**C**) LUC complementation assay showing that JAZ8 inhibits TCP14-ORA interaction. The LUC activities of Cluc-ORA, TCP14-Nluc, and Cluc-Flag were set to 1. The data represent the means ± SD of three replicates from three independent experiments. **P < 0.01, Student’s *t* test. The bottom panel shows a Western blot of proteins isolated from *N. benthamiana* cells. JAZ8-Flag and Cluc-Flag fusion proteins were detected using anti-Flag antibody. Three independent transfection experiments were performed. (**D**) Y2H analysis showing the interactions between ORA, JAZ8, and full-length and truncated versions of TCP14. Left, schematic representations of the truncated TCP14 proteins used in this experiment. Numbers indicate the amino acid (a.a.) positions of the truncated TCP14 variants. The location of the TCP domain is indicated by a yellow box. Right, transformed yeast cells were grown on the selective medium, QDO, and the control medium, DDO, and pictures were taken after 4 days of incubation at 30°C. (**E**) Dual-LUC experiment showing that MeJA treatment partially recovers the activation of the *DBR2* promoter by AaTCP14 and AaORA in the presence of AaJAZ8. The LUC/REN ratio of GFP in the mock treatment was set as 1. Three independent transfection experiments were performed. The data represent the means ± SD of three replicates from three independent experiments. *P < 0.05, **P < 0.01, Student’s *t* test. (**F**) A working model depicting how JA signaling regulates the *DBR2* promoter via interactions between TCP14-ORA and JAZ8. (**a**) In the absence of JA, JAZ8 interacts with both TCP14 and ORA and disrupts the TCP14-ORA complex. This attenuates the transcriptional activation activity of the TCP14-ORA complex at the *DBR2* promoter, decreasing artemisinin biosynthesis. In addition, JAZ8 interacts with MYC2 and may suppress MYC2-mediated artemisinin biosynthesis. (**b**) In the presence of JA, JAZ8 is recognized by COI1 (the JA receptor) and degraded by the 26S proteasome. In the absence of JAZ8, the ORA and TCP14 proteins form a TCP14-ORA complex and synergistically activate the *DBR2* promoter, enhancing artemisinin biosynthesis. In addition, the degradation of JAZ8 releases MYC2, and MYC2 directly or indirectly activates the expression of its target gene *GSW1* and the downstream gene *ORA* to enhance JA-regulated artemisinin biosynthesis. Solid arrow, direct regulation. Broken line arrow, hypothetical direct links. T-bars, negative interactions. 26S, 26S proteasome.

To investigate whether AaJAZ8 regulates the interaction between AaTCP14 and AaORA, we performed Y3H assays. When all three proteins were coexpressed in yeast cells, there was a significant reduction in growth density on selective medium (SD/-T/-L/-H/-A/-M) compared with yeast cells coexpressing only AaTCP14 and AaORA (Fig. 7A). β-Galactosidase (β-gal) activity assays further demonstrated that AaJAZ8 substantially repressed AaTCP14-AaORA interaction (Fig. 7B). These results were independently verified by a competitive LUC complementation assay, where the AaJAZ8-Flag fusion protein was coexpressed with split LUC-tagged AaTCP14 and AaORA proteins in *N. benthamiana* leaf cells. Compared with the Cluc-Flag (Cluc: C-terminal fragment of LUC) negative control, AaJAZ8-Flag significantly reduced the relative LUC activity generated by the interaction between AaTCP14-Nluc and Cluc-AaORA in living cells (Fig. 7C), indicating that AaJAZ8 inhibited the AaTCP14-AaORA interaction.

Next, deletion analysis was performed to map the domain of AaTCP14 that is responsible for the interaction with AaORA and AaJAZ8. Y2H assays showed that truncated versions of AaTCP14 that include the C terminus, TCP14ΔN1 and TCP14ΔN2, interacted with AaORA and AaJAZ8 (Fig. 7D). In contrast, truncated proteins that lacked the C terminus, TCP14ΔC1, TCP14ΔC2, and TCP14MC, were unable to interact with AaORA or AaJAZ8 (Fig. 7D), indicating that the C-terminal domain of AaTCP14 is necessary and sufficient for interaction with AaORA and AaJAZ8. Collectively, these results suggest that AaJAZ8 serves as a competitor of AaORA binding to the C terminus of AaTCP14 and thereby prevents the interaction of AaTCP14 and AaORA.

### AaJAZ8 represses the transcriptional activation activity of the AaTCP14-AaORA complex

It is well known that JAZ repressors have a negative effect on the transcriptional function of their targets (*42*–*44*), and both AaTCP14 and AaORA are targets of AaJAZ8 (Fig. 6). To test whether AaJAZ8 affects the transcriptional activation activity of the AaTCP14-AaORA complex, we performed dual-LUC assays in the presence or absence of MeJA in *N. benthamiana* leaf cells (Fig. 7E). In the absence of MeJA, we found that AaTCP14, AaORA, and AaTCP14-AaORA increased the promoter activity of *DBR2*. However, coexpression with AaJAZ8 significantly decreased the promoter activity of *DBR2* in the presence of all tested combinations of AaTCP14, AaORA, and AaTCP14-AaORA, demonstrating that AaJAZ8 represses the transcriptional activation activity of AaTCP14 and AaORA. Because AaJAZ8 attenuates the interaction between AaTCP14 and AaORA (Fig. 7, A to C), these results support the notion that AaJAZ8 represses the transcriptional activation activity of the AaTCP14-AaORA complex at least in part through inhibiting the interaction between AaTCP14 and AaORA.

In the presence of MeJA, the activation of the *DBR2* promoter by AaTCP14, AaORA, and the AaTCP14-AaORA complex increased. Application of MeJA suppressed the inhibitory effect of AaJAZ8 by triggering its degradation through the 26S proteasome (Fig. 7E and fig. S8C), suggesting that JA can reverse the AaJAZ8-mediated interference with the transcriptional function of AaTCP14 and AaORA. In accordance with these results, the *AaTCP14* overexpression lines had higher levels of JA-induced artemisinin accumulation compared with *A. annua* plants transformed with the empty vector (fig. S10A), while the *AaTCP14* antisense lines had lower levels of JA-induced artemisinin accumulation compared with *A. annua* plants transformed with the empty vector (fig. S10B). These results indicate that the transcriptional function of the AaTCP14-AaORA complex is negatively regulated by AaJAZ8 through its disruption of AaORA and AaTCP14 interaction and also demonstrate that JA mediates artemisinin biosynthesis via the AaORA-AaTCP14 complex.

## DISCUSSION

Malaria is a global health problem, with 216 million new cases and 445,000 deaths observed worldwide in 2016 (*45*). Artemisinin and its derivatives can rapidly kill *Plasmodium* parasites, thereby treating malaria efficiently. However, current artemisinin production is not sufficient to meet global needs. The artemisinin biosynthetic pathways have been well elucidated and involve four key enzymes, ADS, CYP71AV1, DBR2, and ALDH1 (*4*, *5*, *7*–*11*). JA enhances artemisinin content by both elevating the expression levels of these key enzymes and increasing the number of GSTs (*37*). Although JA-responsive transcription factors regulate the activities of *ADS*, *CYP71AV1*, and *DBR2*, the exact mechanism of how these genes interact with the JA pathway is far from being completely understood. Therefore, identification and characterization of JA-responsive protein complexes and transcriptional networks that regulate the expression of these four key enzymes are important for understanding how artemisinin metabolism is regulated. In a previous report, the trichome-specific AP2/ERF transcription factor AaORA was found to be a positive regulator of the *ADS*, *CYP71AV1*, and *DBR2* genes (*22*). However, the mechanism by which AaORA regulates JA-mediated artemisinin biosynthesis has remained unclear. In this study, we identified a novel interactor of AaORA that functions in the regulation of artemisinin biosynthesis. AaTCP14 and AaORA interact in vitro and in vivo. Moreover, AaTCP14 directly binds to and activates the *DBR2* and *ALDH1* promoters. AaTCP14 and AaORA synergistically interact to control the expression of these two genes, and the regulation of *DBR2* and *ALDH1* expression by AaORA is at least partially dependent on AaTCP14. Moreover, the repressor AaJAZ8 interacts with both AaTCP14 and AaORA and disrupts the AaTCP14-AaORA complex, which results in decreased *DBR2* promoter activity. JA induces AaJAZ8 degradation and de-represses the AaTCP14-AaORA complex to promote artemisinin biosynthesis. Thus, we propose that plants have evolved an intricate mechanism for regulation of specialized metabolism through the JA signaling pathway.

AaTCP14 belongs to the class I TCP gene family, members of which are involved in a myriad of plant developmental processes (*29*), hormone signaling (*30*–*32*), and plant metabolism (*35*, *36*). Here, we found that AaTCP14 is JA responsive and directly activates the *DBR2* and *ALDH1* promoters in vivo and in vitro (Figs. 2E and 4). Furthermore, the overexpression and down-regulation of *AaTCP14* activated and repressed, respectively, the expression of artemisinin biosynthetic genes (Fig. 3, B and E), transcription factors in positively regulating artemisinin biosynthesis, and JA biosynthetic genes (fig. S5). The expression levels of these genes may ultimately contribute to the increase and decrease of the artemisinin content in *AaTCP14* overexpression and antisense transgenic plants, respectively (Fig. 3, C and F), indicating that AaTCP14 could be used for engineering artemisinin biosynthesis in *A. annua*.

Notably, AaTCP14 directly binds to the GGTCCCAC or GGCCCCAC sites in the *DBR2* and *ALDH1* promoters, respectively (Fig.4). Both of these sites are consensus binding sites (GGNCCCAC) of class I TCP family transcription factors (*27*, *28*, *33*), suggesting that other members of the class I TCP family may also have the potential to regulate artemisinin biosynthesis by interacting with similar binding sites. Thus, further investigation is needed to determine whether other members of the TCP family are also involved in regulating artemisinin biosynthesis. In addition, we also found a putative class II TBS in the *AaGSW1* promoter (GTGGTCCC, −2098 bp upstream of ATG), which partially overlaps with a putative class I TBS (GGTCC, −2096 bp upstream of ATG), indicating that artemisinin biosynthesis may be coregulated by both class I and class II TCP proteins. In *Arabidopsis*, class I and class II TCPs are known to coregulate SOC1-dependent flowering at multiple levels (*46*). However, the precise mechanism by which class I and class II TCP proteins coregulate artemisinin biosynthesis awaits further investigation.

In plants, numerous cellular functions are often highly coordinated through protein-protein interactions. TCP family members are known to synergistically or antagonistically interact with a variety of proteins to regulate a wide spectrum of biological processes. For example, the ubiquitin receptors DA1, DAR1, and DAR2 modulate AtTCP14/15 stability to regulate endoreduplication in *Arabidopsis* (*47*). AtTCP3 interacts with R2R3-MYB (myeloblastosis) proteins and enhances their transcriptional activation activity, leading to the promotion of flavonoid biosynthesis in *Arabidopsis* (*35*). AtTCP14 and DELLA regulate plant height by regulating GA (gibberellin) signaling in florescence shoot apex in *Arabidopsis* (*32*). In this case, DELLA binds to the DNA binding domain of AtTCP14 and blocks its ability to bind DNA. Intriguingly, we found that AaORA is an important interaction partner of AaTCP14 based on several lines of evidence. First, AaTCP14 and AaORA interact in vitro and in vivo (Fig. 1). In addition, AaTCP14 and AaORA share similar expression patterns (Fig. 2, A and E). Moreover, AaTCP14 and AaORA synergistically regulate the activity of the *DBR2* and *ALDH1* promoters (Fig. 5, A to C) and promote their expression (Fig. 5E). As a result, artemisinin content is higher in *AaTCP14*-*AaORA* co-overexpression lines compared to *AaTCP14* overexpression lines (Fig. 5F). The regulation of *DBR2* and *ALDH1* by AaORA at least partially depends on AaTCP14 (Fig. 5, G to K). Biochemical analysis suggests that AaTCP14 provides the DNA binding ability, while AaORA has strong transcriptional activation activity (Fig. 4 and fig. S1B). Thus, these paired transcription factors act together to increase the artemisinin content in *A. annua* plants.

AaORA has an AP2 DNA binding domain, and its close homolog ORCA3 in *C. roseus* can specifically bind and activate the promoter of the TIA biosynthetic gene *STR* (*strictosidine synthase*) (*23*). In our study, in addition to *DBR2* and *ALDH1*, AaORA also activated the promoters of *ADS* and *CYP71AV1* (fig. S1B). However, AaTCP14 failed to activate the *ADS* or *CYP71AV1* promoters (Fig. 4B). This indicates that AaORA may also regulate artemisinin biosynthesis independently of AaTCP14. Consistent with this notion, we found that additional positive regulators of artemisinin biosynthesis also interact with AaORA in a Y2H screen, suggesting that AaORA might promote artemisinin biosynthesis in *A. annua* through interactions with other proteins. The precise mechanism by which AaORA controls artemisinin biosynthesis will require further investigation.

JA has been found to activate artemisinin production (*37*). Both *AaTCP14* and *AaORA* are JA responsive. Work in *C. roseus* has revealed that CrMYC2 directly activates the promoter of *CrORCA3*, a homolog of *AaORA*, to regulate alkaloid biosynthesis (*25*). Similarly, AaMYC2, a central transcription factor acting downstream of the JA signaling pathway, directly activates the expression of the JA-responsive gene *AaGSW1*, and AaGSW1 activates the expression of *AaORA* and promotes JA-mediated artemisinin biosynthesis (Fig. 7F) (*17*, *18*). Thus, it is possible that AaMYC2 positively regulates *AaORA* expression in both a direct and an indirect manner. The expression levels of *AaMYC2* were up-regulated and down-regulated in *AaTCP14* overexpression and antisense transgenic plants, respectively (fig. S5, B and J), indicating that *AaMYC2* expression is regulated by AaTCP14. This may be a consequence of the regulation of JA biosynthesis by AaTCP14, as AaTCP14 activates the expression of the JA biosynthetic genes *AaAOC* and *AaOPR3* (fig. S5, F and G). Thus, a positive feedback loop between JA signaling and AaTCP14 may allow rapid response to JA for biological processes controlled by AaTCP14, such as artemisinin biosynthesis. Given that most of the abovementioned JA biosynthetic and JA-responsive genes have been reported to promote artemisinin biosynthesis (*17*, *18*, *22*, *38*), AaTCP14 might positively regulate artemisinin biosynthesis by directly or indirectly influencing the JA biosynthesis and signaling pathways.

We uncovered another connection between the AaTCP14-AaORA complex and the JA signaling pathway by identifying AaJAZ8 as an interacting partner for both AaTCP14 and AaORA. JAZ proteins were identified as targets of the SCF^COI1^ complex (*41*) and repress diverse JA-regulated plant responses by interacting with and attenuating the activity of their downstream transcription factors (*42*–*44*). JAZ1 affects the interactions between bHLHs [GL3 (Glabra 3), EGL3 (Enhancer of GL3), and TT8 (Transparent Testa 8)] and MYBs [MYB75 and GL1 (Glabra 1)] proteins by directly interacting with these proteins and thus regulates JA-mediated anthocyanin accumulation and trichome initiation in *Arabidopsis* (*43*). A recent study demonstrated that AtJAZ3 and AtTCP14 do not directly interact, but the *Pseudomonas syringae* type III effector HopBB1 bonds with both AtJAZ3 and AtTCP14 to bring the complex to the proteasome for protein degradation. This process releases the subset of JA responsive genes regulated by AtTCP14 to modulate pathogen virulence in *Arabidopsis* (*48*). Unlike AtJAZ3-HopBB1-AtTCP14, we uncovered direct interactions between AaJAZ8, AaTCP14, and AaORA. AaJAZ8 directly interacts with the AaTCP14-AaORA complex, and this modulates the transcriptional activation of key genes that encode artemisinin biosynthesis enzymes by AaTCP14-AaORA in a JA-responsive manner (Figs. 6 and 7). In JA-elicited cells, AaJAZ8 is degraded, which allows the formation of the AaTCP14-AaORA complex to activate *DBR2* expression (fig. S8C and Fig. 7E). The interference of the AaTCP14-AaORA complex by AaJAZ8 may be due to the competition of binding between AaORA and AaJAZ8 to the C terminus of AaTCP14 (Fig. 7, A to D). Although we found that only AaJAZ8 (out of nine AaJAZ proteins tested) could interact with AaTCP14, we cannot rule out the possibility that these AaJAZ proteins interact with other AaTCP proteins. The JA-responsive regulation of artemisinin biosynthesis reveals a delicate balance wherein plants were involved to cope with the changing environment. Specialized metabolites, such as artemisinin, are already known to affect plant growth when they overaccumulate (*49*); thus, plants need to fine-tune the production of these metabolites, generating the appropriate amount to deal with the biotic stresses with less penalty of growth.

In recent years, it has been found that some JAZ proteins fulfill their functions by forming specific tripartite complexes (*43*, *44*, *50*–*52*). In *Arabidopsis*, AtJAZs repress the transcriptional activation activity of the AtMYC-AtMYB complex by competing with AtMYBs (MYB28, MYB29, MYB76, MYB34, MYB51, and MYB122) for interaction with AtMYCs (MYC2, MYC3, and MYC4) to regulate glucosinolate biosynthesis (*44*). AtJAZs also interact with and repress the AtbHLH-AtMYB complex to control stamen and pollen development (*50*). The AtJAZ-AtMYC-AtMYB and AtJAZ-AtbHLH-AtMYB complexes are similar to the AaJAZ8-AaTCP14-AaORA complex in that JAZs repress the transcriptional activation activities of these complexes to regulate downstream target genes, suggesting that the use of JA signaling to regulate processes, such as specialized metabolism, is a common strategy. Recent work showed that injury induces Ca^2+^/calmodulin-dependent phosphorylation of AtJAV1, which disintegrates the AtJAV1-AtJAZ8-AtWRKY51 complex and de-represses JA biosynthesis in *Arabidopsis* (*51*). This complex is distinct from the AtJAZ-AtMYC-AtMYB, AtJAZ-AtbHLH-AtMYB, and AaJAZ8-AaTCP14-AaORA complexes. In the AtJAV1-AtJAZ8-AtWRKY51 complex, AtJAZ8 enhances the repression of the *AOS* promoter by both AtJAV1 and AtWRKY51, but in the case of AtJAZ-AtMYC-AtMYB, AtJAZ-AtbHLH-AtMYB, and AaJAZ8-AaTCP14-AaORA, JAZ represses the transcriptional activation activity of AtMYC-AtMYB, AtbHLH-AtMYB, and AaTCP14-AaORA, suggesting the common usage of JAZ for different transcriptional regulation purposes.

Do JAZs change the DNA binding ability of their downstream genes? In *Arabidopsis*, DELLAs interact with the AtTCP14 DNA recognition domain and block AtTCP14 activity by keeping it from binding to its targets (*32*). By contrast, AaJAZ8 interacts with the C-terminal region of AaTCP14, and not the DNA recognition domain; hence, it is unlikely that AaJAZ8 alters the DNA binding ability of AaTCP14 (fig. S7C).

On the basis of our current findings, we propose a working model for JA regulation of artemisinin biosynthesis. Briefly, in the absence of JA, AaJAZ8 interacts with both AaTCP14 and AaORA and disrupts the AaTCP14-AaORA complex. This attenuates the transcriptional activation function of AaTCP14-AaORA and decreases activation of the *DBR2* promoter (Fig. 7F), thus impeding artemisinin biosynthesis. By contrast, in the presence of JA, AaJAZ8 is recognized by COI (the JA receptor) and degraded by the 26S proteasome. The released AaTCP14 and AaORA proteins and the AaTCP14-AaORA complex synergistically activate the *DBR2* promoter to enhance artemisinin biosynthesis (Fig. 7F). Given that AaMYC2 positively regulates artemisinin biosynthesis, potentially by activating *AaORA* expression in a direct and indirect manner (*17*, *18*, *25*), and that JAZ repressors, including AaJAZ8, interact with AaMYC2 (fig. S9A), it is possible that AaJAZ8 may function at different transcriptional regulatory layers to fine-tune artemisinin biosynthesis in response to JA signaling. This model provides a foundation for deepening our understanding of the molecular mechanisms of JA regulation of specialized metabolism in plants.

Together, our data indicate that JA enhances artemisinin biosynthesis in *A. annua* through three mechanisms: (i) increasing the transcriptional activation of the *DBR2* promoter by AaTCP14, AaORA, and the AaTCP14-AaORA complex; (ii) degrading the repressor protein AaJAZ8, thereby freeing AaTCP14, AaORA, and the AaTCP14-AaORA complex to activate the transcription of *DBR2*; and (iii) facilitating the formation of AaTCP14-AaORA, which, in turn, enhances *DBR2* promoter activity (Fig. 7F).

## MATERIALS AND METHODS

### Plant materials and hormone treatments

The high-artemisinin cultivar of *A. annua* L., named “Huhao 1,” which originated in Chongqing and was further subjected to several years of selection in Shanghai, was used for all *A. annua*–related assays (*18*). For in vitro culture, seeds of *A. annua* were first surface sterilized in 70% ethanol for 3 min and washed three times with sterile water. This was followed by sterilization in 20% sodium hypochlorite solution for 10 min and then five washes with sterile water. These seeds were sown on Murashige and Skoog medium (Sigma-Aldrich, USA) with 3% sucrose and 0.6% agar (pH 5.7). *A. annua* and *N. benthamiana* plants were grown in pots at 23 ± 2°C under a 16-hour light/8-hour dark photoperiod.

For MeJA treatment, 10-day-old *A. annua* seedlings were sprayed with 100 μM MeJA (Sigma-Aldrich, USA). For the mock treatment, seedlings were sprayed with 0.1% ethanol. Seedling samples were collected at 0, 1, 3, 6, 9, 12, and 24 hours after treatment. To analyze artemisinin content in *AaTCP14* transgenic plants after MeJA treatment, the 2-month-old cutting propagations of *AaTCP14* overexpression, antisense, and control plants (*A. annua* plants transformed with the empty vector) were sprayed with 100 μM MeJA or 0.1% ethanol (mock treatment) and sampled at 48 hours for artemisinin extraction.

### RNA extraction and qRT-PCR

Leaves of 3-month-old *A. annua* at different positions (labeled in Fig. 2B) and different tissues of 4-month-old *A. annua*, including roots, stems, flowers, shoots, buds, leaves, and trichomes, were harvested. To analyze the expression of *TCP14*, *ADS*, *CYP71AV1*, *DBR2*, *ALDH1*, *WRKY1*, *MYC2*, *GSW1*, *ORA*, *AOS*, *AOC*, *OPR3*, and *OPCL1* in *AaTCP14* overexpression lines and *AaTCP14* antisense lines; the expression of *TCP14*, *ORA*, *DBR2*, and *ALDH1* in *AaTCP14-AaORA* co-overexpression lines and *AaTCP14* antisense–*AaORA* overexpression lines; the expression of *ORA*, *DBR2*, and *ALDH1* in *AaORA* overexpression lines; and the expression of *JAZ8*, *ADS*, *CYP71AV1*, *DBR2*, and *ALDH1* in *AaJAZ8* overexpression lines, the leaves of 3-month-old transgenic plants were collected. *A. annua* plants transformed with empty vector (control plants, labeled as vector) and WT plants were used as controls. RNA was extracted using the total plant RNA Extraction Kit (Tiangen Biotech, China) according to the manufacturer’s instructions. cDNA was synthesized from 1.0 μg of total RNA using the PrimeScript 1st Strand cDNA Synthesis Kit (Takara, Japan) according to the manufacturer’s instructions. The expression levels of genes were normalized to the expression of *A. annua Actin*. qRT-PCR analysis was performed as previously described (*22*). All primers used for qRT-PCR are listed in table S1.

### Co-IP assays and degradation of the AaJAZ8 protein

For Co-IP assays with two and three proteins, the full-length coding sequences of *AaTCP14*, *AaORA*, and *AaJAZ8* were cloned from the *A. annua* meristem cDNA library and inserted into the pCambia1300-GFP or pCambia1300-Flag vectors to yield the following constructs: 1300-AaTCP14-GFP, 1300-AaORA-Flag, and 1300-AaJAZ8-Flag with GFP or Flag tag, respectively (primer information is provided in table S1). Five-week-old *N. benthamiana* leaves were transformed by injection of *Agrobacterium* strain GV3101 cells harboring the indicated combinations of 1300-AaORA-Flag, 1300-AaTCP14-GFP, and/or 1300-AaJAZ8-Flag. After incubation at 23°C for 72 hours, the leaves were ground to a fine powder in liquid nitrogen and resuspended in extraction buffer [50 mM tris-HCl (pH 7.5), 150 mM NaCl, 1 mM EDTA (pH 8.0), 0.2% Triton X-100, and protease inhibitors including 100 μM Pefabloc (Sigma-Aldrich, USA), 100 μM cocktail (Roche, Switzerland), and 50 μM MG132 (Calbiochem, USA)]. After incubation on ice for 20 min, the protein suspensions were centrifuged at 14,000 rpm for 10 min. For two-protein Co-IP, the supernatant was incubated with 2 μl of anti-Flag antibody (Sigma-Aldrich, USA) for 1 hour at 4°C. For three-protein Co-IP, the supernatant was incubated with 15 μl of anti-GFP antibody (GenScript, Nanjing, China) for 1 hour at 4°C. Next, 20 μl of prewashed Protein G Sepharose (GE Healthcare, Amersham, UK) was added, and the mixture was incubated for 2 hours at 4°C. Then, the immunoprecipitates were washed four times with extraction buffer and resuspended with 2× SDS sample buffer [500 mM tris-HCl, 12.5% SDS, 25% glycerol, bromophenol blue (0.33 mg/ml), and 10% 2-mercaptoethanol]. Subsequently, the samples were boiled at 100°C for 10 min and centrifuged for 10 s before they were separated by 12% SDS-PAGE (polyacrylamide gel electrophoresis). Proteins were transferred to polyvinylidene fluoride (PVDF) membranes and detected using anti-GFP (Abmart, China) or anti-Flag antibody (Sigma-Aldrich, USA).

To evaluate the degradation of AaJAZ8, 5-week-old *N. benthamiana* leaves were transformed by injection of *Agrobacterium* strain GV3101 cells harboring 1300-AaJAZ8-Flag. After incubation at 23°C for 48 hours, the leaves were pretreated with or without 100 μM MG132 (Calbiochem, USA) for 1 hour and then treated with 50 μM MeJA for 0, 0.5, 1, 2, and 4 hours. The JAZ8-Flag proteins were extracted and detected using anti-Flag antibody (Sigma-Aldrich, USA).

### Dual-LUC assays

For the transient transcriptional activity assays in *N. benthamiana* leaves, the full-length coding sequences of *AaORA*, *AaTCP14*, and *AaJAZ8* were inserted into the pCambia1300-GFP vector (effectors), and the promoter regions upstream of the start codons of *UBQ* (a homolog of *AtUBQ10*) (~1.7 kb), *ADS* (~2.9 kb), *CYP71AV1* (~1.2 kb), *DBR2* (~2.3 kb), and *ALDH1* (~2.3 kb) were ligated into the pGREENII0800-LUC vector (reporters). Infiltration and detection were performed as described previously with a few modifications (*19*). The *Renilla* LUC (REN) gene in pGREENII0800-LUC was used as an internal control. Empty pCambia1300-GFP was used as the negative control for the effector. GV3101 strains harboring indicated combinations of effectors and reporters were cotransformed into 5-week-old *N. benthamiana* leaves. Plants were incubated for 72 hours at 23°C to allow expression of the transgenes. For hormone treatment in dual-LUC assays, *N. benthamiana* leaves were treated with 50 μM MeJA or 0.05% ethanol (mock treatment) for another 4 hours before the sample was collected. For the transient transcriptional activity assays in *A. annua* protoplasts, protoplasts from 14-day-old WT and *AaTCP14* antisense *A. annua* mesophyll cells were prepared and transfected, as previously described (*53*). The reporters and effectors were cotransformed into the WT and *AaTCP14* antisense protoplasts in the indicated combinations, and after transformation, the transformants were cultured in the light for 8 hours. The reporters (*DBR2* and *ALDH1* promoters) were used at 6 μg per transfection, and effectors (GFP and GFP-AaORA) were used at 10 μg per transfection. All experiments were repeated at least three times for each plasmid combination. Firefly LUC and REN activities were analyzed using commercial dual-LUC reaction reagents (Promega, USA) according to the manufacturer’s instructions. The LUC activity was normalized to REN activity, and the relative LUC/REN ratios were used to represent the activity of the promoter. For each combination, LUC/REN ratios from at least three independent transformations were determined. All primers used for these constructs are listed in table S1.

### Y1H assays

For Y1H experiments, the full-length coding sequences of *AaORA*, *AabZIP1*, and *AaTCP14* were amplified using the primers in table S1 and ligated into the pB42AD vector. The promoter sequences of *ADS*, *CYP71AV1*, *DBR2*, and *ALDH1*; three tandem copies of the *E1* motif from *ADS* promoter; the *D1* and *D2* motifs from the *DBR2* promoter; and the *A1*, *A2*, and *A3* motifs from the *ALDH1* promoter were ligated into the pLacZ vector. Various combinations of pB42AD-AabZIP1, pB42AD-AaORA, and pB42AD-AaTCP14 and different promoters and DNA motifs were cotransformed into the yeast strain EGY48. Combinations of pB42AD and different promoters and DNA motifs were also cotransformed into the yeast strain EGY48 as negative controls. The transformants were cultivated on SD/-Trp/-Ura plates, and positive clones were transferred to and grown on SD/-Trp/-Ura plates with X-gal for blue color development. All primers used to amplify promoters and DNA motifs are listed in table S1.

### Y2H assays

The *A. annua* cDNA library for Y2H experiments was constructed by OE BioTech (Shanghai, China) (*42*) by cloning cDNA synthesized from the mRNAs of the youngest leaves and meristem of *A. annua* into the prey vector pGADT7 (Takara, Japan). The full-length coding sequence of *AaORA* and truncated sequences including *AaORA*Δ*C1*, *AaORA*Δ*C2*, *AaORAMC*, *AaORA*Δ*N1*, and *AaORA*Δ*N2* were amplified by PCR using the indicated primers (table S1) and inserted into the bait vector pGKBT7 (Takara, Japan). These recombinant constructs were introduced into the yeast strain AH109 and tested for autoactivation and toxicity. Because of the strong autoactivation of full-length *AaORA*, we used *AaORA*Δ*N1* (C terminus of AaORA) for Y2H screening assays, which were performed according to the Matchmaker Gold Y2H system’s user manual (*Yeast Protocols Handbook*; Takara, Japan).

For additional Y2H experiments to test for specific interactions and to map the protein domain of AaTCP14, AaJAZ8, or AaORA involved in paired interactions, the full-length coding sequences of *AaORA*, *AaMYC2*, and *AaTCP14* and truncated sequences including *AaTCP14*Δ*C1*, *AaTCP14*Δ*C2*, *AaTCP14MC*, *AaTCP14*Δ*N1*, and *AaTCP14*Δ*N2* were amplified using the listed primers (table S1). In addition, the full-length coding sequences of the *JAZ* genes (*AaJAZ1*, *AaJAZ2*, *AaJAZ3*, *AaJAZ4*, *AaJAZ5*, *AaJAZ6*, *AaJAZ7*, and *AaJAZ9*), AaJAZ8, and the truncated variants *AaJAZ8*Δ*ZIM* and *AaJAZ8*Δ*jas* were amplified using the listed primers (table S1). All the aforementioned amplicons were cloned into the pGADT7 or pGKBT7 vectors. Various combinations of plasmids were cotransformed into the yeast strain AH109 according to the manufacturer’s instructions (Takara, Japan), and bait-only or prey-only controls were tested with empty pGADT7 or pGBKT7. The transformants were cultivated on DDO plates, and positive clones were transferred to and grown on TDO and/or QDO plates. Yeast cells were photographed after 4 days of growth at 30°C to record growth. All experiments were repeated three times with similar results.

### Y3H assays

For Y3H assays, the full-length coding sequences of *AaTCP14* and *AaJAZ8* were cloned into the pBridge vector (Takara, Japan) to create DNA binding domain fusion proteins. *AaJAZ8* was inserted into the same pBridge vector driven by the *MET25* promoter to provide Met repression. The full-length coding sequence of *AaORA* was cloned into the pGADT7 vector. Y3H assays were based on the Matchmaker GAL4 three-hybrid systems (Takara, Japan). The combinations pBridge-AaTCP14-**AaJAZ8 and pGADT7-AaORA, and pBridge-AaTCP14 and pGADT7-AaORA were cotransformed into the yeast strain AH109. The recombinant vectors were transformed with empty pGADT7 or pBridge as controls. The transformants were cultivated in liquid SD/-Trp/-Leu media to OD_600_ (optical density at 600 nm) = 0.6 and then diluted to different concentrations. Six-microliter dilutions of suspended yeast were plated on solid medium containing SD/-T/-L/-M and SD/-T/-L/-H/-A/-M. Yeast growth was observed after incubation for 4 days at 30°C. β-Gal assays were performed to quantify protein interaction according to the manufacturer’s instructions (Takara, Japan), using chlorophenol red β-d-galactopyranoside (Roche, Switzerland) as the substrate. The experiments were repeated three times, and the primers used are listed in table S1.

### Electrophoretic mobility shift assays

For protein expression and purification, the full-length coding sequences of *AaORA*, *AaTCP14*, and *AaJAZ8* were cloned into the pCold-TF vector (Takara) to produce His-tagged fusion proteins. The pCold-AaORA, pCold-AaTCP14, and pCold-AaJAZ8 constructs were transferred into *E. coli* strain Rosetta (DE3) (TransGen Biotech, China). The empty pCold-TF vector was introduced into *E. coli* strain Rosetta (DE3) and was used as the negative control. Expression of His fusion proteins in Rosetta cells was induced by adding 0.2 mM isopropyl β-d-1-thiogalactopyranoside (IPTG) to the culture medium and incubating the cells for 14 hours at 16°C. The fusion proteins were purified using Ni-NTA (nitrilotriacetic acid) agarose (Invitrogen, USA) according to the manufacturer’s instructions.

EMSAs were performed as previously described (*19*) with minor modifications using the DIG Gel Shift Kit, 2nd Generation (Roche) according to the manufacturer’s instructions. For these assays, 0.5 μg of recombinant His-TF, 0.5 μg of recombinant His-AaTCP14, and 0.5, 1, 2, and 4 μg of recombinant His-AaORA and His-AaJAZ8 were used. The D2q, D2q-mutated1, D2q-mutated2, A1q, A1q-mutated1, and A1q-mutated2 DNA probes from the *DBR2* and *ALDH1* promoters were synthesized by Sangon (Shanghai, China). The mutated probes were designed as previously described (*33*). The primers and probes used in the EMSAs are listed in table S1.

### BiFC assays

The BiFC assays were performed as previously described (*18*). For the generation of the BiFC vectors, the full-length cDNAs of *AaORA*, *AaTCP14*, and *AaJAZ8* were cloned into pEarleyGate 201-YN (N terminus of YFP) and pEarleyGate 202-YC (C terminus of YFP) to obtain AaORA-cYFP, AaORA-nYFP, AaTCP14-nYFP, and AaJAZ8-cYFP and then transformed into *Agrobacterium* strains GV3101. The indicated vector combinations were cotransformed into 5-week-old *N. benthamiana* leaves. After incubation at 23°C for 60 to 72 hours, YFP signals were observed by confocal laser microscopy (Leica TCS SP5-II). Nuclei were stained with DAPI (Sigma-Aldrich, USA). Three biological repeats were conducted for all experiments. The primers are listed in table S1.

### Subcellular localization and colocalization assays

For subcellular localization experiments, the GV3101 strains harboring pHB-AaTCP14-YFP or pHB-YFP were transformed into 5-week-old *N. benthamiana* leaves. YFP signals were analyzed 60 to 72 hours after infiltration by confocal laser microscopy (Leica TCS SP5-II). Nuclei were stained with DAPI (Sigma-Aldrich, USA). Three biological repeats were performed to verify these results.

For two- and three-protein colocalization assays, the full-length cDNAs of *AaORA*, *AaTCP14*, and *AaJAZ8* were ligated into the pHB-CFP and pHB-YFP vectors to obtain AaORA-CFP, AaTCP14-CFP, AaJAZ8-CFP, and AaJAZ8-YFP. GV3101 strains harboring AaTCP14-CFP and AaJAZ8-YFP, AaORA-CFP and AaJAZ8-YFP, or AaTCP14-nYFP, AaORA-cYFP, and AaJAZ8-CFP were cotransformed into 5-week-old *N. benthamiana* leaves. Meanwhile, GV3101 strains harboring AaORA-CFP and YFP, AaTCP14-CFP and YFP, CFP and AaJAZ8-YFP, or AaTCP14-nYFP, AaORA-cYFP, and CFP were also transformed into 5-week-old *N. benthamiana* leaves as negative controls. After incubation at 23°C for 60 to 72 hours, YFP and CFP signals were observed by confocal laser microscopy (Leica TCS SP5-II). Three biological repeats were performed to verify these results. The primers are listed in table S1.

### In vitro pulldown assays

For protein expression and purification, the full-length coding sequences of *AaORA* and *AaTCP14* were cloned into the pGEX4T-1 (GE Healthcare) and pCold-TF (Takara, Japan) vectors to produce GST-tagged fusion protein and His-tagged fusion protein, respectively. The pGEX4T-1-AaORA and pCold-AaTCP14 constructs were then transferred into *E. coli* strain Rosetta (DE3). Expression of GST and His fusion proteins in Rosetta cells was induced by adding 0.2 mM IPTG to the culture medium and incubating the cells for 14 hours at 16°C. The GST-AaORA and His-AaTCP14 fusion proteins were purified according to the manufacturers’ instructions using glutathione-agarose 4B (GE Healthcare) beads and Ni-NTA agarose (Invitrogen), respectively.

For the pulldown assay, equal amounts (100 μl) of purified GST or GST-AaORA and His-AaTCP14 were mixed and incubated with glutathione-agarose 4B beads for 10 hours at 4°C in 250 μl of pulldown buffer [40 mM Hepes-KOH (pH 7.5), 10 mM KCl, 3 mM MgCl_2_, 0.4 M sucrose, 1 mM EDTA, 1 mM dithiothreitol, 0.2% Triton X-100, and protease inhibitors] with agitation. The beads were then centrifuged at 2000*g* for 1 min at 4°C and washed five times with 1× PBS buffer [137 mM NaCl, 8.1 mM Na_2_HPO_4_⋅12H_2_O, 2.68 mM KCl, 1.47 mM KH_2_PO_4_, and 1 mM phenylmethylsulfonyl fluoride (pH 7.4)]. Bound proteins were eluted by boiling with 5× sample buffer, and the released proteins were separated by 12% SDS-PAGE. Proteins were transferred to PVDF membranes and detected using anti-His (Abmart, China) or anti-GST (Abmart, China) antibody.

### Plant transformation and phenotype analysis

The overexpression constructs pHB-AaTCP14-Flag, 1305-AaORA-GFP, pHB-AaJAZ8, and 1300-AaJAZ8Δjas (a jas-domain deletion version of *AaJAZ8* cDNA); the antisense construct pHB-ANTCP14; the co-overexpression construct 1305-AaTCP14-Myc-AaORA-GFP; and the 1305-AaTCP14 antisense–AaORA overexpression construct were transferred into *Agrobacterium tumefaciens* strain EHA105 and then used to transform *A. annua*, as previously described (*3*). The phenotype of *A. annua* plants transformed with the empty vector (control plants) and *AaTCP14* overexpression, *AaTCP14* antisense, and *AaTCP14*-*AaORA* co-overexpression lines were observed at the indicated times under normal conditions.

### LUC complementation assay

For the LUC complementation assays, the full-length coding sequences of *AaORA* and *AaTCP14* were ligated into pCAMBIA-Nluc and pCAMBIA-Cluc, respectively, and the full-length *AaJAZ8* coding sequence was ligated into pCAMBIA-Cluc. The TCP14-Nluc, ORA-Nluc, Cluc-ORA, and Cluc-JAZ8 constructs were introduced into *A. tumefaciens* strain GV3101. GV3101 strains harboring the indicated constructs were co-infiltrated into *N. benthamiana* leaves, as previously described (*54*).

For the competition LUC complementation assay, GV3101 strains harboring JAZ8-Flag, Cluc-ORA, and TCP14-Nluc were co-infiltrated into *N. benthamiana* leaves. A GV3101 strain harboring Cluc-Flag instead of JAZ8-Flag was co-infiltrated as a control. Leaf discs were taken 3 days later and ground to powder in liquid nitrogen. Then, the relative LUC activities were measured with a LUC kit (Promega, USA) according to the manufacturer’s instructions (Promega, USA). The primers are listed in table S1.

### GUS expression in *1391Z-proTCP14*-*GUS* transgenic *A. annua* plants

To construct *1391Z-proTCP14*-*GUS*, the 1828-bp promoter region upstream of the start codon of *AaTCP14* was amplified with specific primers (table S1) from the *A. annua* genomic DNA library and inserted into the pCambia1391Z vector. Then, the plasmids *1391Z-proTCP14*-*GUS* and *1391Z*-*GUS* were introduced into *A. annua* plants using *Agrobacterium*-mediated genetic transformation, as described previously (*3*). Histochemical staining for GUS activity in transgenic plants was conducted as previously described with minor modifications (*55*). Leaves and stems were stained in a GUS staining solution [1 mM 5-bromo-4-chloro-3-indolyl-β-d-glucuronic acid, 100 mM Na_2_HPO_4_, 50 mM KH_2_PO_4_, 10 mM Na_2_EDTA, 0.5 mM K_3_Fe(CN)_6_, 0.5 mM K_4_Fe(CN)_6_, and 0.1% (v/v) Triton X-100] and incubated at 37°C for 12 to 24 hours in the dark. After GUS staining, 70% ethanol was used to remove chlorophyll. *A. annua* plants transformed with the empty vector were processed in parallel as negative controls.

### Measurement of artemisinin, DHAA, and AA content

Leaves collected from 3-month-old *AaTCP14* transgenic *A. annua* plants; *AaTCP14*-*AaORA* co-overexpression, *AaTCP14* antisense–*AaORA* overexpression, *AaORA* overexpression, and *AaJAZ8* overexpression *A. annua* plants; *A. annua* plants transformed with the empty vector; and WT plants grown in the greenhouse were dried at 50°C overnight and then ground to powder. Dried leaf powder (0.1 g) was extracted twice with 2 ml of methanol under ultrasound for 30 min. Then, the samples were centrifuged for 5 min at 12,000 rpm, and the supernatant was filtered through a nitrocellulose filter (0.22 μM). The concentrations of artemisinin, DHAA, and AA in the final samples were measured by HPLC, as described previously (*22*).

## Supplementary Material

http://advances.sciencemag.org/cgi/content/full/4/11/eaas9357/DC1

## SUPPLEMENTARY MATERIALS

Supplementary material for this article is available at http://advances.sciencemag.org/cgi/content/full/4/11/eaas9357/DC1 Fig. S1. A schematic diagram of the artemisinin biosynthetic pathway and its regulation in *A. annua*, and AaORA activates the *ADS*, *CYP71AV1*, *DBR2*, and *ALDH1* promoters. Fig. S2. Y2H assay showing the regions of AaORA with autoactivation activity. Fig. S3. Alignment of the protein sequences of AaTCP14 and 29 related proteins. Fig. S4. Phylogenetic analysis of TCP14 proteins from *A. annua* and other plants. Fig. S5. Relative expression levels of transcription factors positively regulating artemisinin biosynthesis and JA biosynthetic genes in *AaTCP14* transgenic plants. Fig. S6. Characterization of *A. annua* transgenic plants. Fig. S7. Neither AaORA nor AaJAZ8 affects the ability of AaTCP14 to bind DNA. Fig. S8. The expression patterns of *AaJAZ8*, MeJA-induced AaJAZ8 degradation, and analysis of artemisinin biosynthesis in *A. annua* plants overexpressing *AaJAZ8* or *AaJAZ8*Δ*jas*. Fig. S9. AaTCP14 and AaORA interact with AaJAZ proteins and mapping of the domains involved in the interaction between AaJAZ8, AaORA, and AaTCP14 using Y2H assays. Fig. S10. Artemisinin content in *AaTCP14* transgenic plants under MeJA treatment. Table S1. List of primers used in this study.
