# Supplementary material for: Jasmonate promotes artemisinin biosynthesis by activating the TCP14-ORA complex in Artemisia annua
Source: Sci Adv. 2018 Nov 14;4(11):eaas9357. doi: 10.1126/sciadv.aas9357 (PMC6317983; doi:10.1126/sciadv.aas9357)
Supplement: http://advances.sciencemag.org/cgi/content/full/4/11/eaas9357/DC1 [file supp_4_11_eaas9357__index.html]

Science Advances | Science Advances

## Supplementary Materials

**This PDF file includes:**

- Fig. S1. A schematic diagram of the artemisinin biosynthetic pathway and its regulation in *A. annua*, and AaORA activates the *ADS*, *CYP71AV1*, *DBR2*, and *ALDH1* promoters.
- Fig. S2. Y2H assay showing the regions of AaORA with autoactivation activity.
- Fig. S3. Alignment of the protein sequences of AaTCP14 and 29 related proteins.
- Fig. S4. Phylogenetic analysis of TCP14 proteins from *A. annua* and other plants.
- Fig. S5. Relative expression levels of transcription factors positively regulating artemisinin biosynthesis and JA biosynthetic genes in *AaTCP14* transgenic plants.
- Fig. S6. Characterization of *A. annua* transgenic plants.
- Fig. S7. Neither AaORA nor AaJAZ8 affects the ability of AaTCP14 to bind DNA.
- Fig. S8. The expression patterns of *AaJAZ8*, MeJA-induced AaJAZ8 degradation, and analysis of artemisinin biosynthesis in *A. annua* plants overexpressing *AaJAZ8* or *AaJAZ8*Δ*jas*.
- Fig. S9. AaTCP14 and AaORA interact with AaJAZ proteins and mapping of the domains involved in the interaction between AaJAZ8, AaORA, and AaTCP14 using Y2H assays.
- Fig. S10. Artemisinin content in *AaTCP14* transgenic plants under MeJA treatment.
- Table S1. List of primers used in this study.

Download PDF

**Files in this Data Supplement:**

- Adobe PDF - aas9357\_SM.pdf
