# Supplementary material for: Jasmonate promotes artemisinin biosynthesis by activating the TCP14-ORA complex in Artemisia annua
Source: Sci Adv. 2018 Nov 14;4(11):eaas9357. doi: 10.1126/sciadv.aas9357 (PMC6317983; doi:10.1126/sciadv.aas9357)
Supplement: http://advances.sciencemag.org/cgi/content/full/4/11/eaas9357/DC1 [file aas9357_SM.pdf]

Science Advances
AAAS

advances.sciencemag.org/cgi/content/full/4/11/eaas9357/DC1

Supplementary Materials for

Jasmonate promotes artemisinin biosynthesis by activating the TCP14-ORA complex in *Artemisia annua*

Ya-Nan Ma, Dong-Bei Xu, Ling Li, Fei Zhang, Xue-Qing Fu, Qian Shen, Xue-Ying Lyu, Zhang-Kuanyu Wu, Qi-Fang Pan, Pu Shi, Xiao-Long Hao, Ting-Xiang Yan, Ming-Hui Chen, Pin Liu, Qian He, Li-Hui Xie, Yi-Jun Zhong, Yue-Li Tang, Jing-Ya Zhao, Li-Da Zhang, Xiao-Fen Sun, Ke-Xuan Tang*

*Corresponding author. Email: kxtang@sjtu.edu.cn

Published 14 November 2018, *Sci. Adv.* **4**, eaas9357 (2018)
DOI: 10.1126/sciadv.aas9357

This PDF file includes:

Fig. S1. A schematic diagram of the artemisinin biosynthetic pathway and its regulation in *A. annua*, and AaORA activates the *ADS*, *CYP71AV1*, *DBR2*, and *ALDH1* promoters.
Fig. S2. Y2H assay showing the regions of AaORA with autoactivation activity.
Fig. S3. Alignment of the protein sequences of AaTCP14 and 29 related proteins.
Fig. S4. Phylogenetic analysis of TCP14 proteins from *A. annua* and other plants.
Fig. S5. Relative expression levels of transcription factors positively regulating artemisinin biosynthesis and JA biosynthetic genes in *AaTCP14* transgenic plants.
Fig. S6. Characterization of *A. annua* transgenic plants.
Fig. S7. Neither AaORA nor AaJAZ8 affects the ability of AaTCP14 to bind DNA.
Fig. S8. The expression patterns of *AaJAZ8*, MeJA-induced AaJAZ8 degradation, and analysis of artemisinin biosynthesis in *A. annua* plants overexpressing *AaJAZ8* or *AaJAZ8△jas*.
Fig. S9. AaTCP14 and AaORA interact with AaJAZ proteins and mapping of the domains involved in the interaction between AaJAZ8, AaORA, and AaTCP14 using Y2H assays.
Fig. S10. Artemisinin content in *AaTCP14* transgenic plants under MeJA treatment.
Table S1. List of primers used in this study.

SUPPLEMENTARY MATERIALS

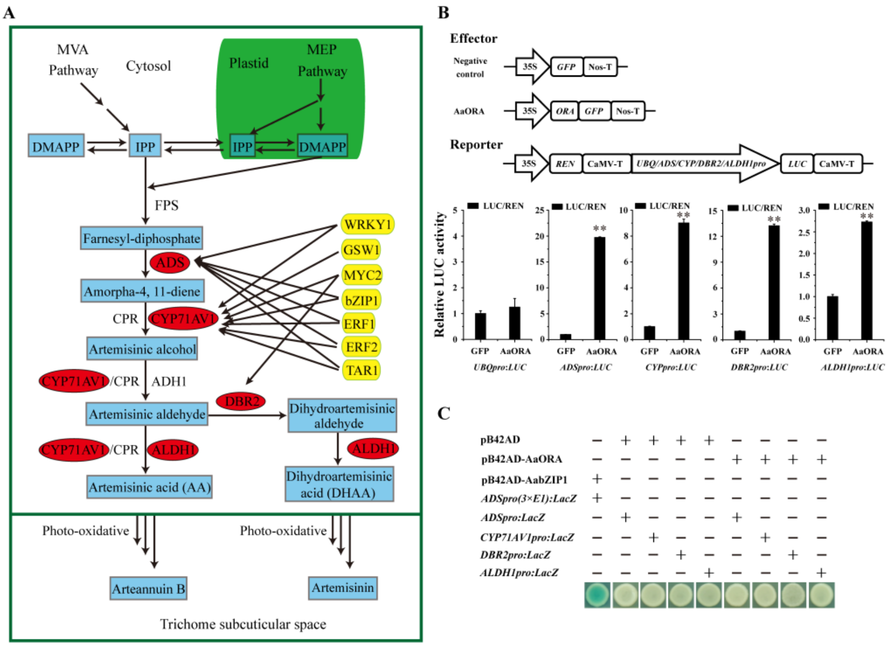

Fig. S1. A schematic diagram of the artemisinin biosynthetic pathway and its regulation in *A. annua*, and AaORA activates the *ADS*, *CYP71AV1*, *DBR2*, and *ALDH1* promoters. (**A**) Biosynthetic pathways and regulation of artemisinin in *Artemisia annua*. MVA, mevalonate; MEP, 2-C-methyl-D-erythritol 4-phosphate; IPP, isopentenyl diphosphate; DMAPP, dimethylallyl diphosphate; FPS, farnesyl diphosphatesynthase; ADS, amorpha-4,11-diene synthase; CYP71AV1, cytochrome P450 monooxygenase; CPR, cytochrome P450 reductase; ADH1, alcohol dehydrogenase 1; DBR2, artemisinic aldehyde∆11(13) reductase; ALDH1, aldehyde dehydrogenase 1; WRKY1, WRKY transcription factor; GSW1, Glandular Trichome-Specific WRKY1; MYC2, basic helix-loop-helix (bHLH) transcription factor; bZIP1, basic leucine zipper transcription factor; ERF1 and ERF2, ETHYLENE-RESPONSIVE FACTOR 1 and 2; TAR1, TRICHOME AND ARTEMISININ REGULATOR1. (**B**) Top, Schematic diagrams of the effector (AaORA-GFP) and reporter (35S: REN-*UBQ/ADS*/*CYP*/*DBR2*/*ALDH1*$_{pro}$:LUC) plasmids used for the Dual-LUC assays. CYP, CYP71AV1. REN, Renilla luciferase. LUC, firefly luciferase. Bottom, Dual-LUC experiments showing the activation of the *UBQ*, *ADS*, *CYP71AV1*, *DBR2* and *ALDH1* promoters by ORA in *N. benthamiana* leaves. GFP was used as a negative control, and the LUC/REN ratios of GFP were set as 1. Three independent transfection

experiments were performed. The data represent the means ± SD of three replicates from three independent experiments. **$P < 0.01$, Student's *t*-test. (**C**) Yeast one-hybrid assay showing that AaORA dose not bind to the *ADS*, *CYP71AV1*, *DBR2* and *ALDH1* promoters. The combination of pB42AD-AabZIP1 and *ADSpro(3×E1):LacZ* was used as a positive control. Yeast cells co-expressing pB42AD, pB42AD-AaORA or pB42AD-AabZIP1 and the full-length *ADS*, *CYP71AV1*, *DBR2* or *ALDH1* promoters or the DNA motif from the *ADS* promoter were grown on selective medium, SD/-Trp/-Ura, containing 20 mg/L X-gal, and pictures were taken after 4 days of incubation at 30°C. Blue plaques indicate protein-DNA interactions. The Y1H assays were repeated three times, and representative results are shown.

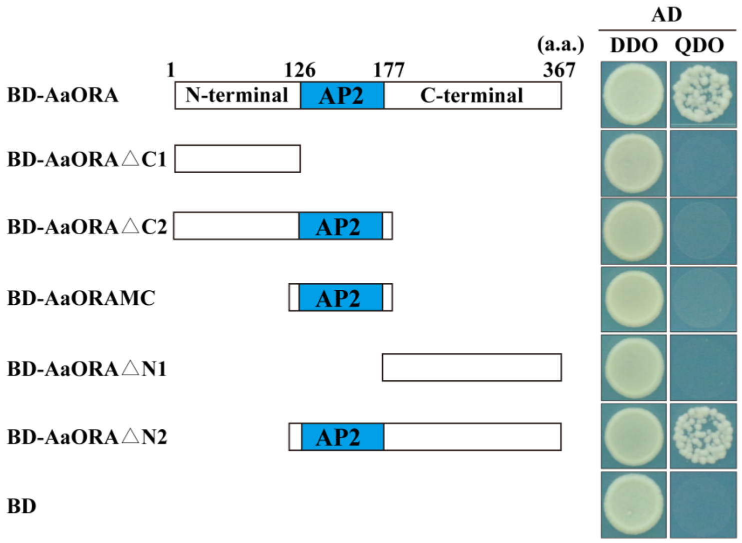

Fig. S2. Y2H assay showing the regions of AaORA with autoactivation activity. Left, schematic representations of the full-length and truncated AaORA proteins used in this experiment. Numbers indicate the amino acid positions of the truncated AaORA variants. The AP2 domain is indicated by a blue box. Right, Y2H assays of the interactions between AD and truncated versions of BD-AaORA. Transformed yeast cells were grown on selective medium, SD/-Trp/-Leu/-His/-Ade (QDO), and control medium, SD/-Trp/-Leu (DDO), and pictures were taken after 4 days of incubation at 30°C. The Y2H assays were repeated three times, and representative results are shown.

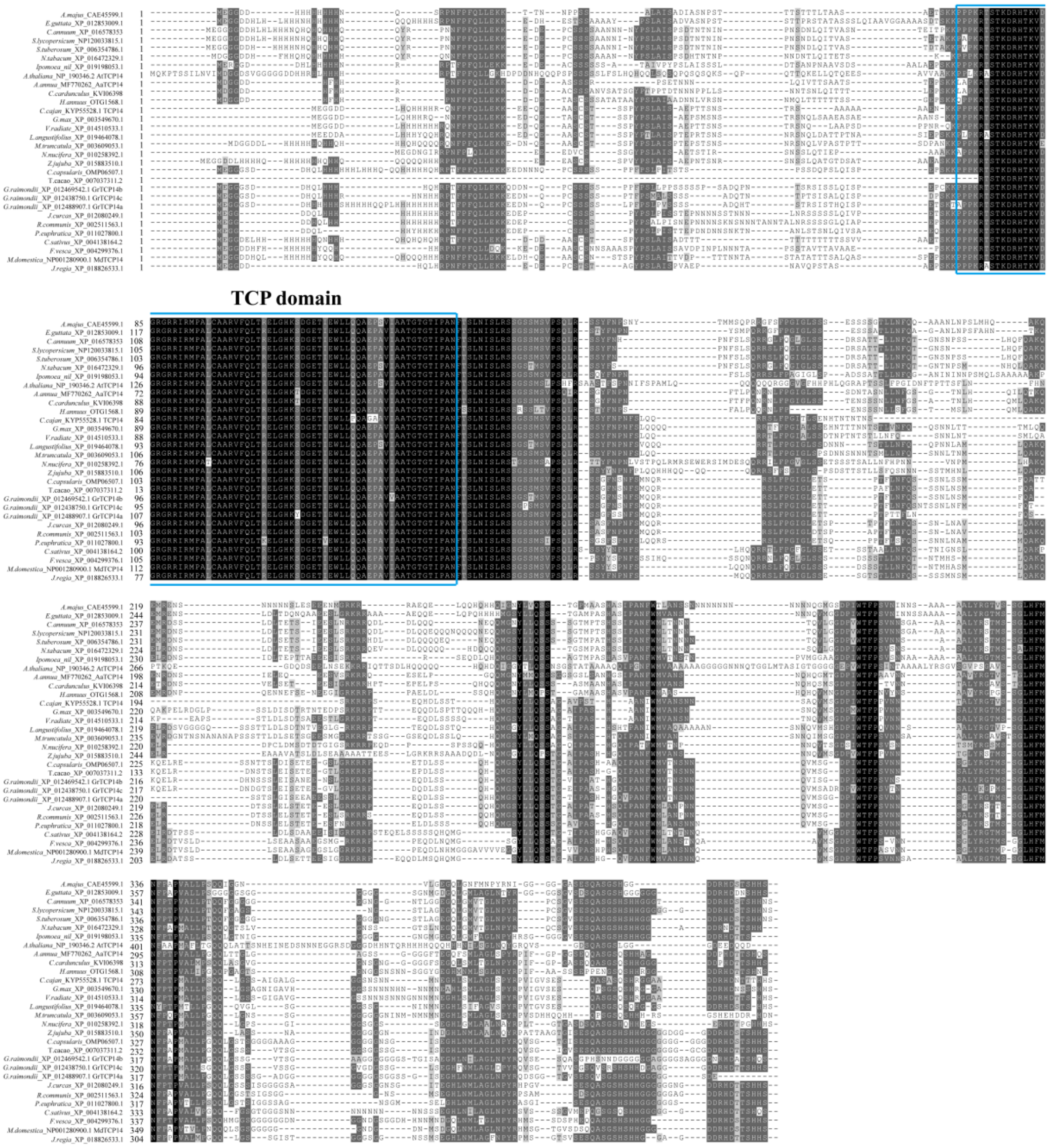

Fig. S3. Alignment of the protein sequences of AaTCP14 and 29 related proteins.

Alignment of the amino acid sequences of AaTCP14 and other TCP14 proteins. Identical amino acids are shaded in black, conserved residues are shaded in dark grey and similar residues are shaded in light grey. The conserved TCP domains are outlined with a blue box. The accession numbers of the sequences shown are as follows: CAE45599.1 from *Antirrhinum majus*; XP_012853009.1 from *Erythranthe guttata*; XP_016578353 from *Capsicum annuum*; NP120033815.1 from *Solanum lycopersicum*; XP_016472329.1 from *Nicotiana tabacum*; XP_019198053.1 from *Ipomoea nil*; AT3G47620_AtTCP14 from *Arabidopsis thaliana*; MF770262_AaTCP14 from *Artemisia annua*; KVI06398 from *Cynara cardunculus*; OTG1568.1 from *Helianthus annuus*; KYP55528.1 from *Cajanus cajan*;

XP_003549670.1 from *Glycine max*; XP_014510533.1 from *Vigna radiate*; XP_019464078.1 from *Lupinus angustifolius*; XP_003609053.1 from *Medicago truncatula*; XP_010258392.1 from *Nelumbo nucifera*; XP_015883510.1 from *Ziziphus jujube*; OMP06507.1 from *Corchorus capsularis*; XP_007037311.2 from *Theobroma cacao*; XP_012488907.1_GrTCP14a, XP_012469542.1_GrTCP14b and XP_012438750.1_GrTCP14c from *Gossypium raimondii*; XP_012080249.1 from *Jatropha curcas*; XP_002511563.1 from *Ricinus communis*; XP_011027800.1 from *Populus euphratica*; XP_004138164.2 from *Cucumis sativus*; XP_004299376.1 from *Fragaria vesca*; NP001280900.1 from *Malus domestica*; XP_018826533.1 from *Juglans regia*.

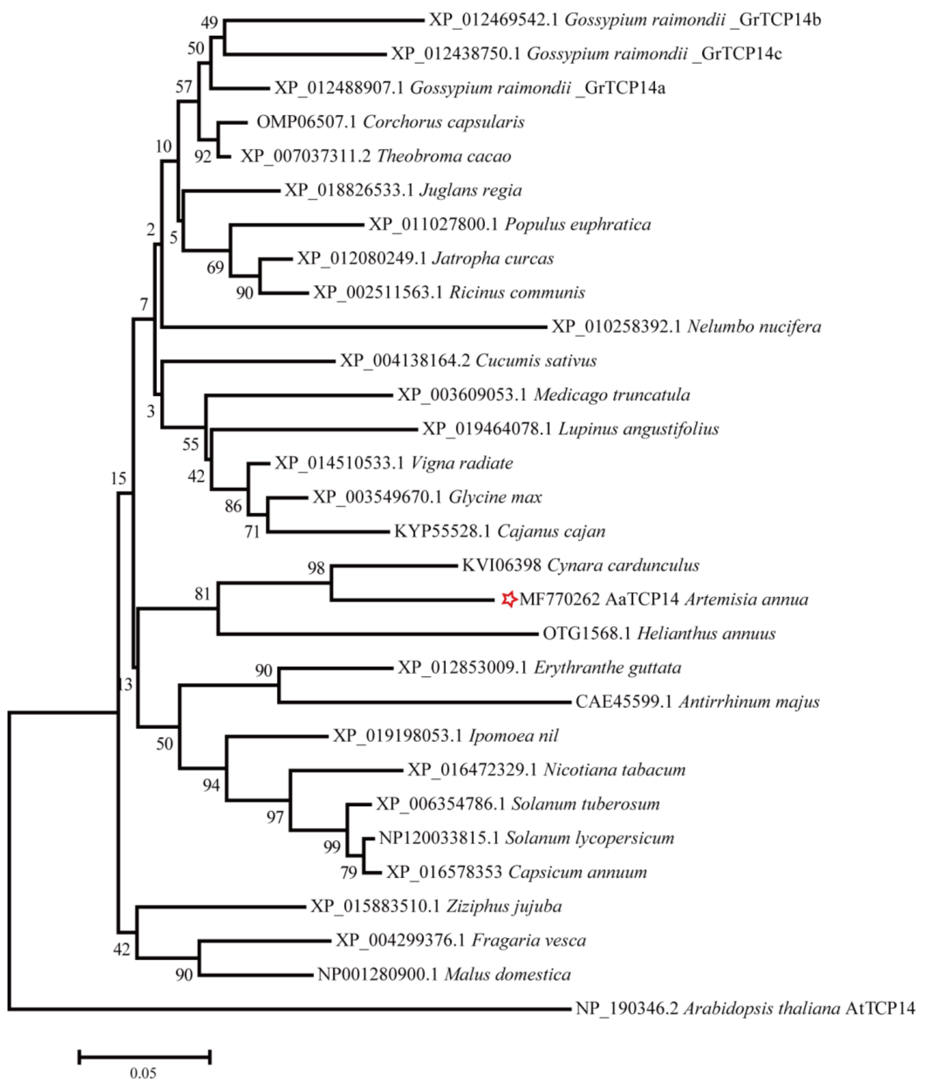

Fig. S4. Phylogenetic analysis of TCP14 proteins from *A. annua* and other plants. TCP14 sequences from different plant species were aligned with Clustal W, and a neighbor-joining phylogenetic tree was constructed using MEGA. Bootstrap values indicate the percentage of 1000 replicates. AaTCP14 is marked with a red star. The amino acid sequences are named based on the NCBI accession number and species name.

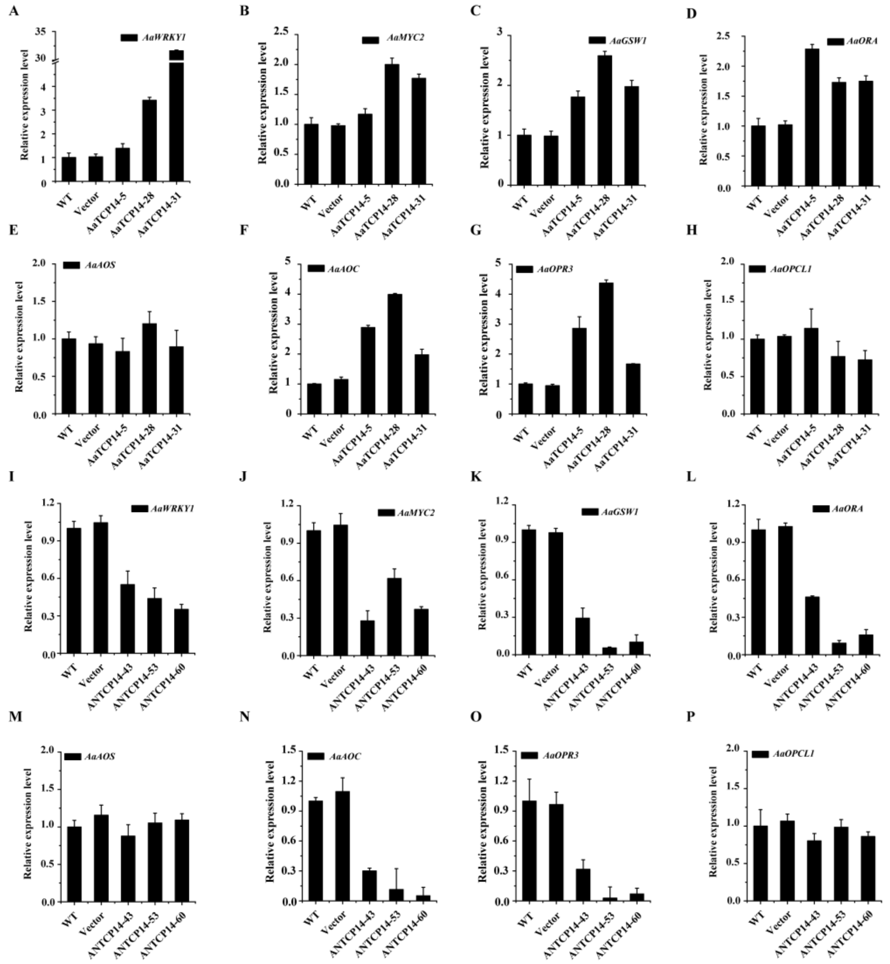

Fig. S5. Relative expression levels of transcription factors positively regulating artemisinin biosynthesis and JA biosynthetic genes in *AaTCP14* transgenic plants. (**A-P**) The expression levels of *AaWRKY1* (A, I), *AaMYC2* (B, J), *AaGSW1* (C, K), *AaORA* (D, L), *AaAOS* (E, M), *AaAOC* (F, N), *AaOPR3* (G, O) and *AaOPCL1* (H, P) in 3-month-old *A. annua AaTCP14*-overexpression and antisense (ANTCP14) plants, plants transformed with the empty vector (Vector) and wild-type (WT) were determined by quantitative real-time PCR (qRT-PCR). *Actin* was used as an internal control. The data represent the means ± SD of three replicates from three cutting propagations.

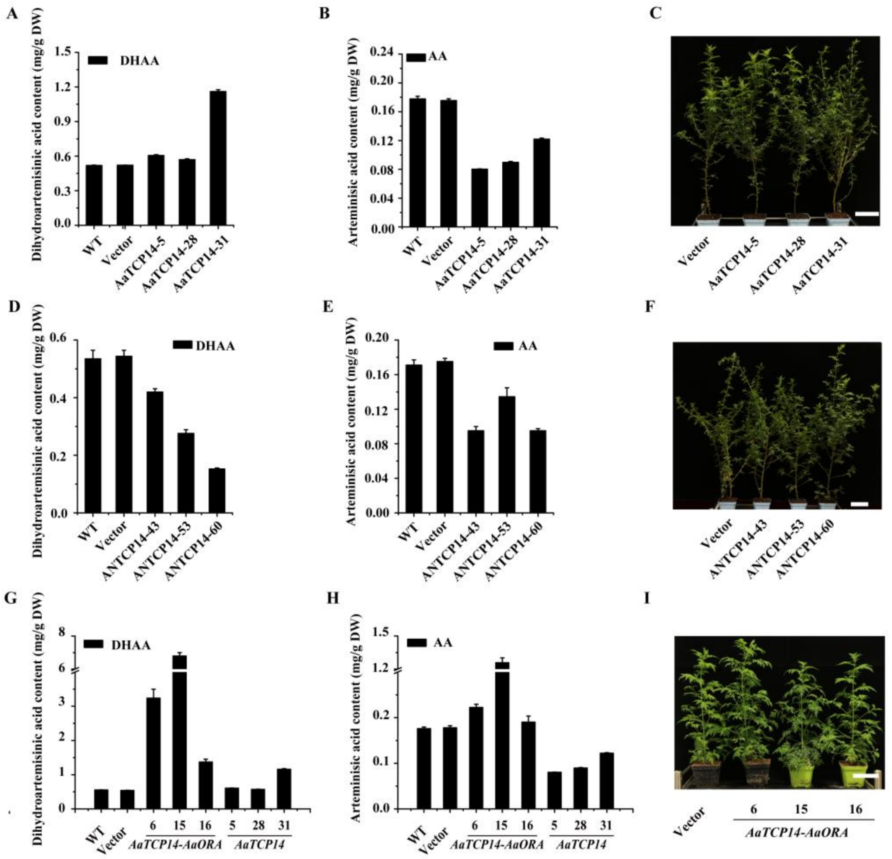

Fig. S6. Characterization of *A. annua* transgenic plants. (**A**, **B**, **D**, **E**, **G**, **H**) HPLC analysis of dihydroartemisinic acid (DHAA) and artemisinic acid (AA) in the leaves of different *A. annua AaTCP14*-overexpression lines (A and B), *AaTCP14*-antisense lines (D and E), *AaTCP14-AaORA* co-overexpression lines (G and H), plants transformed with the empty vector (Vector) and WT plants. The data represent the means ± SD of three replicates from three cutting propagations. (**C** and **F**) The phenotypes of 3-month-old *A. annua* plants transformed with the empty vector (control plants, labled as Vector), three independent *AaTCP14*-overexpression lines (C) and *AaTCP14*-antisense lines (F). (**I**) The phenotypes of 2-month-old *A. annua* plants transformed with the empty vector (control plants, labeled as Vector) and three independent *AaTCP14-AaORA* co-overexpression (*AaTCP14-AaORA*) lines. Scale bar = 10 cm.

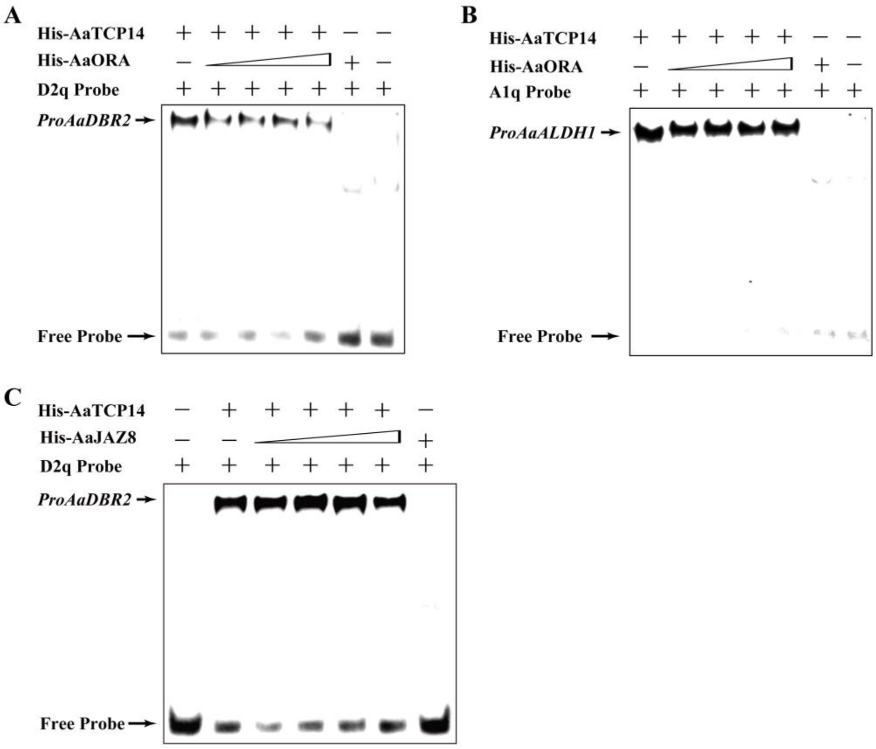

Fig. S7. Neither AaORA nor AaJAZ8 affects the ability of AaTCP14 to bind DNA. (**A-C**) EMSA assay of AaTCP14 binding to the *D2* motif from the *DBR2* promoter (A, C), and the *A1* motif from the *ALDH1* promoter (B). Binding reactions contained 0.5 μg of His-AaTCP14 protein and an increasing amount (0.5 μg, 1 μg, 2 μg, 4 μg) of His-AaORA or His-AaJAZ8 protein. The labeled D2q and A1q probes are the same as in Fig. 4.

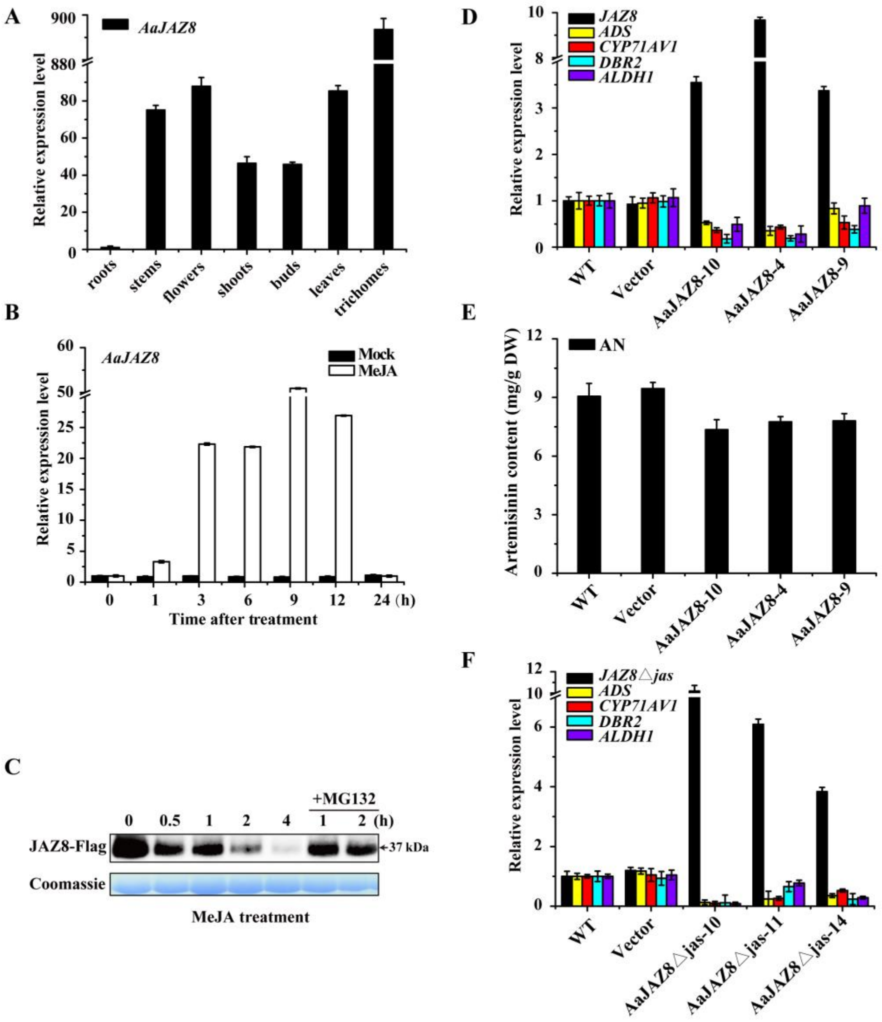

Fig. S8. The expression patterns of *AaJAZ8*, MeJA-induced AaJAZ8 degradation, and analysis of artemisinin biosynthesis in *A. annua* plants overexpressing *AaJAZ8* or *AaJAZ8*∆*jas*. (**A**) Relative expression levels of *AaJAZ8* in different tissues, including roots, stems, flowers, shoots, buds, leaves and trichomes, were measured by quantitative real-time PCR. The expression level of *AaJAZ8* in roots was set as 1. *Actin* was used as an internal control. The data represent the means ± SD from three replicates from three independent *A. annua* plants. (**B**) The expression levels of *AaJAZ8* in plants treated with 100 μM MeJA or 0.1% ethanol (Mock) over 24 hours. *Actin* was used as an internal control. The data represent the means ± SD of three replicates from three independent *A. annua* plants. (**C**) MeJA stimulates AaJAZ8 turnover in a 26S proteasome-dependent manner. *N. benthamiana* leaves expressing JAZ8-Flag were pretreated with or without the 26S proteasome inhibitor MG132 (100 μM) for 1 hour, and then the leaves were treated with MeJA (50 μM) for 0, 0.5, 1, 2, and 4 hours.

Western blot analysis with anti-Flag antibody was done to measure the levels of JAZ8-Flag. A Coomassie-stained SDS-PAGE gel (Coomassie) was used to confirm equal sample loading. (**D**) Expression levels of *JAZ8*, *ADS*, *CYP71AV1*, *DBR2* and *ALDH1* in different *A. annua AaJAZ8*-overexpression lines, plants transformed with the empty vector (Vector) and WT. *Actin* was used as the internal control. The data represent the means ± SD from three replicates from three cutting propagations. (**E**) HPLC analysis of artemisinin (AN) in the leaves of *AaJAZ8*-overexpression lines, plants transformed with the empty vector (Vector) and WT plants. The data represent the means ± SD of three replicates from three cutting propagations. (**F**) Expression levels of *JAZ8⊿jas*, *ADS*, *CYP71AV1*, *DBR2* and *ALDH1* in different *A. annua AaJAZ8⊿jas*-overexpression lines, plants transformed with the empty vector and WT. *Actin* was used as the internal control. The data represent the means ± SD from three technical replicates.

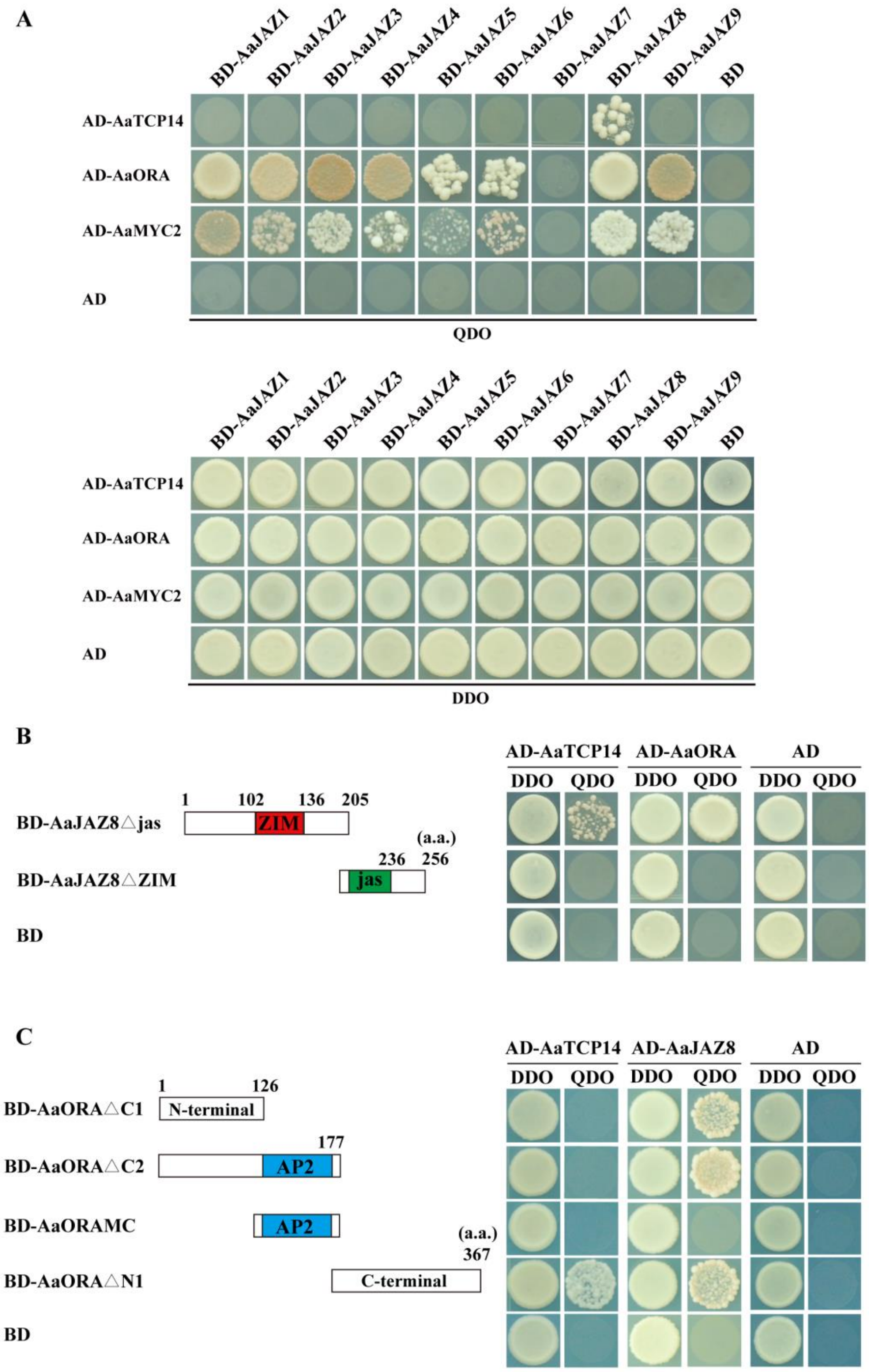

Fig. S9. AaTCP14 and AaORA interact with AaJAZ proteins and mapping of the domains involved in the interaction between AaJAZ8, AaORA, and AaTCP14 using Y2H assays. (**A**) Interactions between AaJAZ proteins and AaTCP14, AaORA or AaMYC2 in Yeast two hybrid (Y2H) assays. (**B**) Y2H assays showing the interactions between AaTCP14, AaORA and truncated versions of AaJAZ8. Left, schematic representations of the truncated AaJAZ8 protein used in this experiment. Numbers indicate the amino acid positions of the

truncated AaJAZ8 variants. The ZIM and jas domains are indicated by the red box and green box, respectively. Right, Y2H assays of protein interactions between AD-TCP14, AD-ORA and truncated versions of BD-JAZ8. (**C**) Y2H assays showing the interactions between AaTCP14, AaJAZ8 and truncated versions of AaORA. Left, schematic representations of the truncated AaORA protein used in this experiment. Numbers indicate the amino acid positions of the truncated AaORA variants. The AP2 domain is indicated by a blue box. Right, Y2H assays of protein interactions between AD-TCP14, AD-JAZ8 and truncated versions of BD-ORA. Transformed yeast cells were grown on the selective medium QDO and control medium DDO, and pictures were taken after 4 days of incubation at 30°C. The Y2H assays were repeated three times, and representative results are shown.

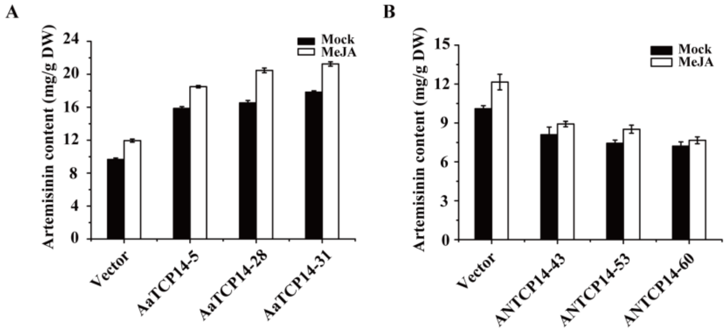

Fig. S10. Artemisinin content in *AaTCP14* transgenic plants under MeJA treatment. (**A** and **B**) Artemisinin content in *A. annua AaTCP14*-overexpression lines (A), *AaTCP14*-antisense lines (B) and plants transformed with the empty vector (control plants, labeled as Vector) was measured by HPLC after 48 h of treatment with 100 μM MeJA or 0.1% ethanol (Mock). The data represent the means ± SD of three replicates from three cutting propagations.

Table S1. List of primers used in this study.

**Name**	**Primer sequence (5’-3’)**
**Primers for the Dual-LUC assay**	
1300-AaORA-GFP-F	GGGGCCCGGGGTCGACATGTTTGCTACTTGCATTCG
1300-AaORA-GFP-R	TACCGGATCCACTAGTAAAAAAAAAAAAGTCATCAT
1300-AaTCP14-GFP-F	GGGGCCCGGGGTCGACATGGATGGTGGTGATGATCA
1300-AaTCP14-GFP-R	TACCGGATCCACTAGTCGACTGATGGCTAGTTGTAT
1300-AaJAZ8-GFP-F	GGGGCCCGGGGTCGACATGGAGAGGGATTTTATGGG
1300-AaJAZ8-GFP-R	TACCGGATCCACTAGTCTTGCTTGCAACAGCGATAG
pGreen0800-UBQ-F	CGGTATCGATAAGCTTGCGGATACAATTTCAATATA
pGreen0800-UBQ-R	ATCCCCCGGGCTGCAGGGTCTTACCAGTGAGGGTCT
pGreen0800-ADS-F	CGGTATCGATAAGCTTCTCGAGTATGGTGTTTCAAC
pGreen0800-ADS-R	ATCCCCCGGGCTGCAGGATTTTCAAAACTTTGAATA
pGreen0800-CYP-F	CGGTATCGATAAGCTTAATGGGTCAATTTCGGGTTG
pGreen0800-CYP-R	ATCCCCCGGGCTGCAGCATGCTTTTAGTATACTC
pGreen0800-DBR2-F	CGGTATCGATAAGCTTAAGAACTTCGAGATAGAAAA
pGreen0800-DBR2-R	ATCCCCCGGGCTGCAGTCAGTGATGGAGTTGGTAAA
pGreen0800-ALDH1-F	CGGTATCGATAAGCTTATGAACCATTAGAAGGGAAG
pGreen0800-ALDH1-R	ATCCCCCGGGCTGCAGCTTTGTTTTTTATGAAATTT
**Primers for constructs used in the Y1H assay**	
pB42AD-AaORA-F	TGCCTCTCCCGAATTCATGTTTGCTACTTGCATTCG
pB42AD-AaORA-R	TCCAAAGCTTCTCGAGTCAAAAAAAAAAAAAAGTCAT
pB42AD-AabZIP1-F	TGCCTCTCCCGAATTCATGAACTACAAGAATTTTGG
pB42AD-AabZIP1-R	TCCAAAGCTTCTCGAGTCACCATGGACCGGAAAGTG
pB42AD-AaTCP14-F	TGCCTCTCCCGAATTCATGGATGGTGGTGATGATCA
pB42AD-AaTCP14-R	TCCAAAGCTTCTCGAGTCACGACTGATGGCTAGTTG
ADSpro:LacZ-F	TATTGGATCGGAATTCCTCGAGTATGGTGTTTCAAC
ADSpro:LacZ-R	GAGCACATGCCTCGAGGATTTTCAAAACTTTGAATA
CYP71AV1pro:LacZ-F	TATTGGATCGGAATTCAATGGGTCAATTTCGGGTTG
CYP71AV1pro:LacZ-R	GAGCACATGCCTCGAGCATGCTTTTAGTATACTC
DBR2pro:LacZ-F	TATTGGATCGGAATTCAAGAACTTCGAGATAGAAAA
DBR2pro:LacZ-R	GAGCACATGCCTCGAGTCAGTGATGGAGTTGGTAAA
ALDH1pro:LacZ-F	TATTGGATCGGAATTCATGAACCATTAGAAGGGAAG
ALDH1pro:LacZ-R	GAGCACATGCCTCGAGCTTTGTTTTTTATGAAATTT
DBR2pro(3×D1):LacZ-F	AATTCCACGGTCCCACTTTCACGGTCCCACTTTCACGGTCCCACTTTC
DBR2pro(3×D1):LacZ-R	TCGAGAAAGTGGGACCGTGAAAGTGGGACCGTGAAAGTGGGACCGTGG
DBR2pro(3×D2):LacZ-F	AATTCTAAGCCCAAATTAAGCCCAAATTAAGCCCAAATC
DBR2pro(3×D2):LacZ-R	TCGAGATTTGGGCTTAATTTGGGCTTAATTTGGGCTTAG
ALDH1pro(3×A1):LacZ-F	AATTCTCTGGCCCCACCGTTCTGGCCCCACCGTTCTGGCCCCACCGTC
ALDH1pro(3×A1):LacZ-R	TCGAGACGGTGGGGCCAGAACGGTGGGGCCAGAACGGTGGGGCCAGAG
ALDH1pro(3×A2):LacZ-F	AATTCCCAGCCCAATACCAGCCCAATACCAGCCCAATAC
ALDH1pro(3×A2):LacZ-R	TCGAGTATTGGGCTGGTATTGGGCTGGTATTGGGCTGGG
ALDH1pro(3×A3):LacZ-F	AATTCTTAGCCCAATATTAGCCCAATATTAGCCCAATAC

ALDH1pro(3×A3):LacZ-R	TCGAGTATTGGGCTAATATTGGGCTAATATTGGGCTAAG
ABI5pro(3×B1):LacZ-F	AATTCCATGGGTCCCACACACCATGGGTCCCACACACCATGGGTCCCACACACC
ABI5pro(3×B1):LacZ-R	TCGAGGTGTGTGGGACCCATGGTGTGTGGGACCCATGGTGTGTGGGACCCATGG
**Primers used to generate DNA constructs for the Y2H assay**	
BD-AaORA-F	GGAGGACCTGCATATGATGTTTGCTACTTGCATTCG
BD-AaORA-R	GGATCCCCGGGAATTCTCAAAAAAAAAAAAAAGTCAT
BD-AaORA-ΔC1-F	GGAGGACCTGCATATGATGTTTGCTACTTGCATTCG
BD-AaORA-ΔC1-R	GGATCCCCGGGAATTCTCAACGTCTCCTGACTCCTC
BD-AaORA-ΔC2-F	GGAGGACCTGCATATGATGTTTGCTACTTGCATTCG
BD-AaORA-ΔC2-R	GGATCCCCGGGAATTCTCAACAGCCTATTAAGAGAG
BD-AaORA-ΔN1-F	GGAGGACCTGCATATGTTAATAGGCTGTGATGATCG
BD-AaORA-ΔN1-R	GGATCCCCGGGAATTCTCAAAAAAAAAAAAAAGTCAT
BD-AaORA-ΔN2-F	GGAGGACCTGCATATGGTCAGGAGACGTCCGTGGGG
BD-AaORA-ΔN2-R	GGATCCCCGGGAATTCTCAAAAAAAAAAAAAAGTCAT
BD-AaORA-MC-F	GGAGGACCTGCATATGGTCAGGAGACGTCCGTGGGG
BD-AaORA-MC-R	GGATCCCCGGGAATTCTCAACAGCCTATTAAGAGAG
BD-AaTCP14-F	GGAGGACCTGCATATGATGGATGGTGGTGATGATCA
BD-AaTCP14-R	GGATCCCCGGGAATTCTCACGACTGATGGCTAGTTG
BD-AaTCP14-ΔC1-F	GGAGGACCTGCATATGATGGATGGTGGTGATGATCA
BD-AaTCP14-ΔC1-R	GGATCCCCGGGAATTCTCATTTTTTCGAGGGTTCTGTTG
BD-AaTCP14-ΔC2-F	GGAGGACCTGCATATGATGGATGGTGGTGATGATCA
BD-AaTCP14-ΔC2-R	GGATCCCCGGGAATTCTCAATTTAAGGAAGTGAAGTTAG
BD-AaTCP14-ΔN1-F	GGAGGACCTGCATATGACTTCCTTAAATATTTCACT
BD-AaTCP14-ΔN1-R	GGATCCCCGGGAATTCTCACGACTGATGGCTAGTTG
BD-AaTCP14-ΔN2-F	GGAGGACCTGCATATGCCCTCGAAAAAACTAGCTCC
BD-AaTCP14-ΔN2-R	GGATCCCCGGGAATTCTCACGACTGATGGCTAGTTG
BD-AaTCP14-MC-F	GGAGGACCTGCATATGCCCTCGAAAAAACTAGCTCC
BD-AaTCP14-MC-R	GGATCCCCGGGAATTCTCAATTTAAGGAAGTGAAGTTAG
BD-AaJAZ8-F	GGAGGACCTGCATATGATGGAGAGGGATTTTATGGG
BD-AaJAZ8-R	GGATCCCCGGGAATTCTCACTTGCTTGCAACAGCGA
BD-AaJAZ8-ΔZIM-F	GGAGGACCTGCATATGGGACAAGCGATGCAATCAGC
BD-AaJAZ8-ΔZIM-R	GGATCCCCGGGAATTCTCACTTGCTTGCAACAGCGA
BD-AaJAZ8-Δjas-F	GGAGGACCTGCATATGATGGAGAGGGATTTTATGGG
BD-AaJAZ8-Δjas-R	GGATCCCCGGGAATTCTCATTGCATCGCTTGTCCGA
BD-AaJAZ1-F	GGAGGACCTGCATATGATGTCGATGGCTAGAAACTT
BD-AaJAZ1-R	GGATCCCCGGGAATTCCTATACGTTGAGATCAAATT
BD-AaJAZ2-F	GGAGGACCTGCATATGATGTCATCGGCTAAACAATT
BD-AaJAZ2-R	GGATCCCCGGGAATTCCTATAAATTTAGATCGAACT
BD-AaJAZ3-F	GGAGGACCTGCATATGATGGAAAGGGATTTCATGGG
BD-AaJAZ3-R	GGATCCCCGGGAATTCTCACTTTGTATCATCTTTGC
BD-AaJAZ4-F	GGAGGACCTGCATATGATGGGTTCATCAGAAATTGT
BD-AaJAZ4-R	GGATCCCCGGGAATTCTTATTGGACATGAGATTGTG
BD-AaJAZ5-F	GGAGGACCTGCATATGATGTCTGAGACTGTGGATTC

BD-AaJAZ5-R	GGATCCCCGGGAATTCTTATTGGGCACCAGAAGATT
BD-AaJAZ6-F	GGAGGACCTGCATATGATGTCGGCTGCCCAACGTTT
BD-AaJAZ6-R	GGATCCCCGGGAATTCTTACATATTAAGGTCGAAAC
BD-AaJAZ7-F	GGAGGACCTGCATATGATGGAACGAGATTTTATGGG
BD-AaJAZ7-R	GGATCCCCGGGAATTCCTAATGCTCGCCTGCTGCTA
BD-AaJAZ9-F	GGAGGACCTGCATATGATGTTGAGATCACCCTCGGT
BD-AaJAZ9-R	GGATCCCCGGGAATTCTCAATGCAAAGACAAGTCTC
AD-AaTCP14-F	AGATTACGCTCATATGATGGATGGTGGTGATGATCA
AD-AaTCP14-R	CACCCGGGTGGAATTCTCACGACTGATGGCTAGTTG
AD-AaORA-F	TGCCTCTCCCGAATTCATGTTTGCTACTTGCATTCG
AD-AaORA-R	TCCAAAGCTTCTCGAGTCAAAAAAAAAAAAAGTCAT
AD-AaJAZ8-F	AGATTACGCTCATATGATGGAGAGGGATTTTATGGG
AD-AaJAZ8-R	CACCCGGGTGGAATTCTCACTTGCTTGCAACAGCGA
AD-AaMYC2-F	AGATTACGCTCATATGATGACGATGAATATATGGAA
AD-AaMYC2-R	CACCCGGGTGGAATTCTTACCTAGGATCTGACATTC
**Primers for constructs in pull-down assay**	
pCold-AaTCP14-F	CGAAGGTAGGCATATGATGGATGGTGGTGATGATCA
pCold-AaTCP14-R	AGATTACCTATCTAGATCACGACTGATGGCTAGTTG
pGEX4T-1-AaORA-F	GGTTCCGCGTGGATCCATGTTTGCTACTTGCATTCG
pGEX4T-1-AaORA-R	GTCGACCCGGGAATTCTCAAAAAAAAAAAAAGTCAT
**Primers for constructs used in the BiFC experiment**	
Topo-AaTCP14-F	CACCATGGATGGTGGTGATGATCA
Topo-AaTCP14-R	CGACTGATGGCTAGTTGTAT
Topo-AaORA-F	CACCATGTTTGCTACTTGCATTCG
Topo-AaORA-R	AAAAAAAAAAAAGTCATCAT
Topo-AaJAZ8-F	CACCATGGAGAGGGATTTTATGGG
Topo-AaJAZ8-R	CTTGCTTGCAACAGCGATAG
**Primers for constructs used in the Co-IP assays and degradation assay**	
1300-AaORA-Flag-F	GGGGCCCGGGGTCGACATGTTTGCTACTTGCATTCG
1300-AaORA-Flag-R	TACCGGATCCACTAGTAAAAAAAAAAAAGTCATCAT
1300-AaJAZ8-Flag-F	GGGGCCCGGGGTCGACATGGAGAGGGATTTTATGGG
1300-AaJAZ8-Flag-R	TACCGGATCCACTAGTCTTGCTTGCAACAGCGATAG
**Primers for constructs used in the subcellular localization experiment**	
pHB-AaTCP14-YFP-F	CGAGCTGCAGGAGCTCATGGATGGTGGTGATGATCA
pHB-AaTCP14-YFP-R	TGCTCACCATACTAGTCGACTGATGGCTAGTTGTATC
**Primers for analyzing genes expression**	
Actin-F	CCAGGCTGTTCAGTCTCTGTAT
Actin-R	CGCTCGGTAAGGATCTTCATCA
QPCR-AaTCP14-F	AGGGACGGTATCTACGGGTT
QPCR-AaTCP14-R	AGCGTGATGCGATTGAGA
QPCR-antisense-AaTCP14-F	GCTTCTCTCTCTCTTCACAT
QPCR-antisense-AaTCP14-R	GTGGCAGCACTTGTAGTTA
QPCR-ADS-F	GGACTAGGTTCAGGCTATG

QPCR-ADS-R	GGACTAGGTTCAGGCTATG
QPCR-CYP71AV1-F	TCATTTCAGTCGCTT
QPCR-CYP71AV1-R	CCAGTTTGCCTCAGTA
QPCR-DBR2-F	ACTGCTGGTGGCTTTCTTA
QPCR-DBR2-R	ACCCTCGACTTGTTCCTTA
QPCR-ALDH1-F	GGACTTGCCTCAGGTGTAT
QPCR-ALDH1-R	GTGCCTCTAATCCTTGTTC
QPCR-AaORA-F	TCCTCCTCTTCATCCTCTATCG
QPCR-AaORA-R	CGTTGTCCTTTCTGTCCTCAGT
QPCR-AaJAZ8-F	ATGGTCCCTTTATGGCAGC
QPCR-AaJAZ8-R	AATGATGCTTTGCGAGCC
QPCR-AaJAZ8∆jas-F	CATTCGGTTCTTCCATCTACC
QPCR-AaJAZ8∆jas -R	CTGCCATAAAGGGACCATTT
QPCR-AaMYC2-F	TGAGGAAGTTACTGATACAGAATGG
QPCR-AaMYC2-R	GTATTCCGAATACCTGACCTTGT
QPCR-AaGSW1-F	TCTCGTCAAAGACACACACATTC
QPCR-AaGSW1-R	TTGTTCGTAGTTGCTGTAGTGCT
QPCR-AaWRKY1-F	TAGCGTTGATGATGGTTACA
QPCR-AaWRKY1-R	ACAGGTGGCGAATAGACT
QPCR-AaAOS-F	CAACTACTACCACAACCACTACTGG
QPCR-AaAOS-R	TGTCGGGAAAGATTTAGCG
QPCR-AaAOC-F	GTCCTGCTTACCTACGGTTGGG
QPCR-AaAOC-R	TTCTGTCGTGATGTATGCGCCT
QPCR-AaOPR3-F	ATGGTCAAACAGAAGCGGG
QPCR-AaOPR3-R	TGTCAAGAGAAGGGTAATCCG
QPCR-AaOPCL1-F	ACAACCTCCACATTCCACAG
QPCR-AaOPCL1-R	GACGGATTCAACGGCTTT
**Primers for constructs used in the EMSA assays**	
pCold-AaORA-F	CGAAGGTAGGCATATGATGTTTGCTACTTGCATTCG
pCold-AaORA-R	AGATTACCTATCTAGATCAAAAAAAAAAAAAAGTCAT
pCold-AaJAZ8-F	CGAAGGTAGGCATATGATGGAGAGGGATTTTATGGG
pCold-AaJAZ8-R	AGATTACCTATCTAGATCACTTGCTTGCAACAGCGA
D2q Probe-F	GTCGCTAAACTTGCCACGGTCCCACTTTTTCAACTCTTATTA
D2q Probe-R	TAATAAGAGTTGAAAAAGTGGGACCGTGGCAAGTTTAGCGAC
D2q Probe-mutant1-F	GTCGCTAAACTTGCCACGGTAAACATTTTTCAACTCTTATTA
D2q Probe-mutant1-R	TAATAAGAGTTGAAAAATGTTTACCGTGGCAAGTTTAGCGAC
D2q Probe-mutant2-F	GTCGCTAAACTTGCCACGTTGAAACATTTTTCAACTCTTATTA
D2q Probe-mutant2-R	TAATAAGAGTTGAAAAATGTTTCAACGTGGCAAGTTTAGCGAC
A1q Probe-F	GGTTGGTTGCATTATCTGGCCCCACCGTAAACAATCAAATGC
A1q Probe-R	GCATTTGATTGTTTACGGTGGGGCCAGATAATGCAACCAACC
A1q Probe-mutant1-F	GGTTGGTTGCATTATCTGGCAAACACGTAAACAATCAAATGC
A1q Probe-mutant1-R	GCATTTGATTGTTTACGTGTTTGCCAGATAATGCAACCAACC
A1q Probe-mutant2-F	GGTTGGTTGCATTATCTTTAAAACACGTAAACAATCAAATGC

A1q Probe-mutant2-R	GCATTTGATTGTTTACGTGTTTTAAAGATAATGCAACCAACC
**Primers for constructs used to generate the of AaTCP14, AaTCP14-AaORA, ANTCP14-AaORA, AaJAZ8, AaJAZ8∆jas and 1391Z-proTCP14 transgenic plants**	
pHB- AaTCP14-Flag-F	GGATCCTACCTGCAGATGGATGGTGGTGATGATCA
pHB- AaTCP14-Flag-R	AACGAAAGCTCTAGACGACTGATGGCTAGTTGTATC
pHB- ANTCP14-F	CGAGCTGCAGGAGCTCCGACTGATGGCTAGTTGTAT
pHB- ANTCP14-R	TGCTCACCATACTAGTATGGATGGTGGTGATGATCA
1305-AaTCP14-Myc-F	GGACTCTTGACCATGGTAATGGATGGTGGTGATGATCA
1305-AaTCP14-Myc-R	ATTCGAGCTGGTCACCTTATACCGAGTTCAAGTCCT
1305-AaORA-GFP-F	GACTCTTGACCATGGTAATGTTTGCTACTTGCATTCG
1305-AaORA-GFP-R	ATTCGAGCTGGTCACCTTACTTGTACAGCTCGTCCA
1305-ANTCP14-GFP-F	GGACTCTTGACCATGGTACGACTGATGGCTAGTTGTAT
1305-ANTCP14-GFP-R	ATTCGAGCTGGTCACCTTACTTGTACAGCTCGTCCA
pHB-AaJAZ8-F	CGAGCTGCAGGAGCTCATGGAGAGGGATTTTATGGG
pHB-AaJAZ8-R	TGCTCACCATACTAGTCTTGCTTGCAACAGCGATAG
pHB-AaJAZ8∆jas-YFP-F	CGAGCTGCAGGAGCTCATGGAGAGGGATTTTATGGG
pHB-AaJAZ8∆jas-YFP-R	TGCTCACCATACTAGTTTGCATCGCTTGTCCGAGTG
1391Z-proTCP14-F	CCAAGCTTGGCTGCAGGCATTGACCACTGAGATGACAGT
1391Z-proTCP14-R	GAATTCCCGGGGATCCAGAGAGATAACTAGGCGTGATTG
**Primers for constructs used in Co-localization experiments**	
pHB-AaJAZ8-YFP-F	CGAGCTGCAGGAGCTCATGGAGAGGGATTTTATGGG
pHB-AaJAZ8-YFP-R	TGCTCACCATACTAGTCTTGCTTGCAACAGCGATAG
pHB-AaORA-CFP-F	GCAGCCCGGGGGATCCATGTTTGCTACTTGCATTCG
pHB-AaORA-CFP-R	TGCTCACCATACTAGTAAAAAAAAAAAAGTCATCAT
pHB- AaTCP14-CFP-F	GCAGCCCGGGGGATCCATGGATGGTGGTGATGATCA
pHB- AaTCP14-CFP-R	TGCTCACCATACTAGTCGACTGATGGCTAGTTGTATC
**Primers for constructs used in Luciferase complementation assays**	
Cluc-JAZ8-F	GTCCCGGGGCGGTACCATGGAGAGGGATTTTATGGG
Cluc-JAZ8-R	AGCTCTGCAGGTCGACTCACTTGCTTGCAACAGCGA
TCP14-Nluc-F	GGACGAGCTCGGTACCATGGATGGTGGTGATGATCA
TCP14-Nluc-R	ACGAGATCTGGTCGACCGACTGATGGCTAGTTGTATC
ORA-Nluc-F	GGACGAGCTCGGTACCATGTTTGCTACTTGCATTCG
ORA-Nluc-R	ACGAGATCTGGTCGACAAAAAAAAAAAAGTCATCAT
Cluc-AaORA-F	GTCCCGGGGCGGTACCATGTTTGCTACTTGCATTCG
Cluc-AaORA-R	AGCTCTGCAGGTCGACTCAAAAAAAAAAAAAAGTCAT
**Primers for constructs used in the Y3H assay**	
pBridge(BD)-AaTCP14-F	TGTATCGCCGGAATTCATGGATGGTGGTGATGATCA
pBridge(BD)-AaTCP14-R	GGCTGCAGGTCGACGTCACGACTGATGGCTAGTTG
pBridge(Met)-AaJAZ8-F	AAAGGTGGCGGCCGCAATGGAGAGGGATTTTATGGG
pBridge(Met)-AaJAZ8-R	ATCAGCCCGAAGATCTTCACTTGCTTGCAACAGCGA
